# An Integrative Transcriptomic, Network Pharmacology, and Molecular Docking Analysis of the Ferroptosis–Fibrosis Axis in Cardiomyopathy with Exploratory Relevance to Diabetic Cardiomyopathy

**DOI:** 10.3390/biomedicines14071501

**Published:** 2026-07-02

**Authors:** Lutfi Cagatay Onar, Ersin Guner, Ibrahim Yilmaz

**Affiliations:** 1 Department of Cardiovascular Surgery, Dr. Ismail Fehmi Cumalioglu City Hospital, Ministry of Health, Republic of Turkey, Tekirdag 59020, Türkiye; 2 Department of Pharmacy, Konya Numune Hospital, Ministry of Health, Republic of Turkey, Konya 42060, Türkiye; 3 Unit of Pharmacovigilance, Dr. Ismail Fehmi Cumalioglu City Hospital, Ministry of Health, Republic of Turkey, Tekirdag 59020, Türkiye

**Keywords:** cardiac fibrosis, cardiomyopathy, extracellular matrix remodeling, ferroptosis, machine learning, molecular docking, network pharmacology, transcriptomic analysis

## Abstract

**Background:** Diabetic cardiomyopathy (DCM) is characterized by metabolic dysfunction, inflammation, extracellular matrix (ECM) remodeling, and myocardial fibrosis. Increasing evidence suggests that ferroptosis-associated oxidative injury may contribute to cardiac remodeling; however, the interaction between ferroptosis-related pathways and fibrosis-associated molecular networks remains incompletely understood. This study explored the ferroptosis–fibrosis axis using an integrative transcriptomic and systems pharmacology framework. **Methods:** Differentially expressed genes were identified from the GSE5406 myocardial transcriptomic dataset comparing nonfailing donor hearts with ischemic and idiopathic cardiomyopathy samples and analyzed using functional enrichment, protein–protein interaction, and disease-association approaches. Cross-dataset comparison and exploratory sample-level external evaluation were performed using the independent GSE263297 DCM-related dataset. Candidate genes were further evaluated by receiver operating characteristic (ROC) analysis and machine learning-based feature selection using least absolute shrinkage and selection operator (LASSO), random forest, and support vector machine-recursive feature elimination (SVM-RFE). Representative compounds associated with fibrosis-, oxidative stress-, inflammation-, and ferroptosis-related pathways were subsequently assessed by molecular docking against TGFBR1, STAT3, GPX4, AKT1, SMAD3, and ACSL4. **Results:** Transcriptomic analyses highlighted ECM organization, collagen-containing ECM, and fibrosis-related pathways as dominant biological themes. Cross-dataset comparison showed partial preservation of transcriptional patterns between independent myocardial cohorts, with 20 of 51 evaluated genes demonstrating concordant expression direction across datasets. ROC analysis identified *LUM* and *ASPN* as having the highest area under the curve (AUC) values among candidate genes, whereas *COL1A1*, *COL1A2*, and *COL3A1* also showed elevated AUC values. Machine learning analyses identified *FCN3*, *HOPX*, *CNN1*, and *GLUL* as the core signature consistently prioritized across all three algorithms, whereas *LUM* was additionally identified by two of three algorithms. Internal validation yielded a cross-validated AUC of 0.934 (95% CI: 0.820–1.000), and exploratory sample-level external evaluation of the four-gene signature in GSE263297 yielded an AUC of 0.673 (95% CI: 0.380–0.967). Exploratory docking analyses suggested potential structural compatibility between several candidate compounds and fibrosis-, inflammation-, and ferroptosis-associated targets, with comparatively lower predicted binding-energy values observed for selected ligand–target combinations. **Conclusions:** The findings are consistent with a fibrosis-dominant remodeling signature and suggest potential network-level links between ferroptosis-associated processes and cardiac fibrosis. These observations should be regarded as exploratory and hypothesis-generating and require validation in independent cohorts and experimental studies.

## 1. Introduction

Diabetic cardiomyopathy (DCM) is increasingly recognized as a distinct myocardial disorder characterized by structural remodeling and functional impairment in the absence of overt coronary artery disease or uncontrolled hypertension [1]. The clinical phenotype encompasses early diastolic dysfunction, progressive myocardial stiffness, and, in advanced stages, systolic impairment. These alterations are thought to arise from a complex interplay of metabolic derangements, oxidative stress, and chronic low-grade inflammation associated with diabetes mellitus [2].

At the cellular level, hyperglycemia-driven metabolic stress promotes mitochondrial dysfunction, excessive reactive oxygen species (ROS) generation, and impaired redox homeostasis [2]. These changes not only disrupt cardiomyocyte energetics but also activate maladaptive signaling pathways that contribute to both cell death and extracellular matrix (ECM) remodeling. In this context, increasing attention has been directed toward regulated forms of cell death beyond apoptosis, particularly ferroptosis, which is characterized by iron-dependent lipid peroxidation [3].

Ferroptosis is mechanistically linked to the failure of antioxidant defense systems, most notably involving glutathione peroxidase 4 (GPX4), which normally detoxifies lipid hydroperoxides [3]. Impairment of GPX4 activity, coupled with increased availability of redox-active iron and enhanced incorporation of polyunsaturated fatty acids into membrane phospholipids via acyl-CoA synthetase long-chain family member 4 (ACSL4), creates a permissive environment for lipid peroxidation and membrane damage [3,4]. Emerging experimental data suggest that these processes may contribute to cardiomyocyte injury in diabetic settings, although their precise role in human DCM remains to be fully elucidated [2,3].

Parallel to cell death mechanisms, myocardial fibrosis represents a central structural hallmark of DCM and a major determinant of adverse clinical outcomes [5]. Fibrotic remodeling is largely driven by activation of the transforming growth factor-beta (TGF-β) signaling pathway, leading to downstream SMAD-dependent transcriptional programs that promote ECM deposition and fibroblast activation [5]. The resulting increase in myocardial stiffness contributes directly to diastolic dysfunction and impaired ventricular compliance.

Importantly, fibrotic processes do not occur in isolation but are closely modulated by inflammatory and metabolic signaling networks. Among these, signal transducer and activator of transcription 3 (STAT3) and protein kinase B (AKT1) have been implicated as key integrative nodes linking metabolic stress, inflammation, and tissue remodeling [6]. STAT3 activation has been associated with both pro-inflammatory and profibrotic responses, while AKT1 signaling plays a dual role in cell survival and metabolic adaptation under stress conditions [6]. These pathways may therefore serve as critical mediators bridging cellular injury and structural remodeling in DCM.

Taken together, these observations are compatible with the concept that ferroptosis and fibrosis may represent interconnected rather than independent processes within the diabetic heart. Oxidative stress, mitochondrial dysfunction, and inflammatory signaling constitute plausible mechanistic links that integrate these pathways into a broader ferroptosis–fibrosis axis. However, despite growing interest in this concept, current evidence remains fragmented, with most studies focusing on individual pathways rather than their integrated network behavior.

From a therapeutic perspective, the multifactorial nature of DCM poses a challenge for conventional single-target interventions. Increasing emphasis has therefore been placed on multitarget pharmacological strategies capable of modulating multiple interconnected pathways simultaneously. In this setting, computational approaches such as network pharmacology provide a systems-level framework to identify potential regulatory hubs and drug–target interactions across complex biological networks [7]. When integrated with molecular docking techniques, these approaches may offer additional structural insights into ligand–target binding affinities and interaction patterns.

Nevertheless, it should be emphasized that such computational strategies are inherently exploratory and hypothesis-generating. They do not establish causality but rather provide a structured means of prioritizing targets and candidate compounds for further experimental validation. Accordingly, careful interpretation and methodological transparency are essential to avoid overstatement of findings.

In this context, the present study was designed as an integrative systems-level investigation of the ferroptosis–fibrosis axis in cardiomyopathy with exploratory relevance to DCM. Publicly available transcriptomic data were analyzed to identify differentially expressed genes, characterize enriched biological processes and pathways, construct protein–protein interaction networks, and prioritize candidate hub genes associated with ECM remodeling and stress-related responses. To provide additional support for candidate gene prioritization, receiver operating characteristic (ROC) analysis and machine learning–based feature selection approaches were subsequently applied. Finally, molecular docking was used as an exploratory structural approach to evaluate potential ligand–target compatibility within selected fibrosis-, ferroptosis-, and stress-related pathways.

The primary objective of this study was not to establish causal mechanisms or therapeutic efficacy, but rather to explore potential biological relationships linking ECM remodeling, ferroptosis-associated stress responses, and cardiometabolic dysfunction within an integrated analytical framework. The resulting observations are intended to generate testable hypotheses, prioritize candidate genes and pathways for future investigation, and provide a foundation for subsequent experimental validation.

## 2. Materials and Methods

### 2.1. Dataset and Study Design

Gene expression data were obtained from the publicly available Gene Expression Omnibus (GEO) database under accession number GSE5406 [8]. The GSE5406 dataset was originally generated and reported in a transcriptomic study of human heart failure [9]. This dataset comprises human left ventricular myocardial tissue samples collected from nonfailing donor hearts and patients diagnosed with cardiomyopathy [8]. For the purposes of the present analysis, samples were stratified into two groups based on clinical status: nonfailing myocardial samples were designated as the control group, whereas samples from patients with ischemic and idiopathic cardiomyopathy were combined and analyzed as the disease group. This grouping strategy was adopted to increase statistical power and to capture shared transcriptional alterations associated with cardiomyopathic remodeling. The control group consisted of 16 nonfailing donor hearts, whereas the disease group comprised 194 cardiomyopathy samples, including 108 ischemic and 86 idiopathic cardiomyopathy cases.

An independent transcriptomic dataset (GSE263297) was additionally retrieved from the GEO database and used for exploratory cross-dataset comparison in order to assess the directional consistency of the identified gene expression patterns. All analyses were conducted exclusively on Homo sapiens samples, and no cross-species integration was performed in order to maintain biological and translational consistency. The present study represents a secondary analysis of publicly available transcriptomic data. All datasets used in this study are publicly available, and no additional ethical approval was required. For GSE263297, log2 fold-change values were recalculated from normalized expression data by comparing ICM-DM samples with donor controls. Statistical comparisons were performed using Welch’s *t*-test, and *p*-values were adjusted using the Benjamini–Hochberg false discovery rate method. These analyses were performed solely to inform cross-dataset directional comparison and were not used for differential gene selection. The original publication associated with GSE263297 was also reviewed to ensure appropriate biological interpretation of the independent cohort [10].

In addition to the directional cross-dataset comparison, a sample-level external evaluation was performed using the normalized log2-transformed RNA-sequencing expression matrix provided within GSE263297. Expression values of the machine-learning-derived core signature genes *FCN3*, *HOPX*, *CNN1*, and *GLUL* were extracted from the independent cohort. A composite four-gene score was calculated for each sample as the mean of standardized expression values across the four genes. Receiver operating characteristic (ROC) analysis was then performed to evaluate the ability of this four-gene score to distinguish ICM-DM samples from donor myocardial samples. The area under the curve (AUC) and corresponding 95% confidence interval (CI) were calculated using the DeLong method. This analysis was conducted as an exploratory sample-level external evaluation of the machine-learning–derived signature and was not used for gene selection.

### 2.2. Differential Expression Analysis

Differential expression analysis was conducted using the GEO2R web tool (accessed on 20 May 2026) provided by the NCBI Gene Expression Omnibus (GEO), which is implemented based on the limma package from the Bioconductor project [11,12]. This approach applies linear models combined with empirical Bayes moderation to improve variance estimation and statistical reliability [13]. Gene expression values were log2-transformed when required to ensure comparability across samples, and probes with missing values were excluded prior to analysis. Samples were assigned into control and disease groups as described above, and a design matrix was constructed to model group-specific effects. In the GEO2R output, positive logFC values indicate higher expression in nonfailing control samples relative to cardiomyopathy samples, whereas negative logFC values indicate higher expression in cardiomyopathy samples relative to nonfailing control samples. Statistical significance was determined using moderated t-statistics with *p*-values adjusted for multiple testing using the Benjamini–Hochberg false discovery rate (FDR) method [14,15]. Differentially expressed genes (DEGs) were defined using a threshold of adjusted *p*-value < 0.05 and an absolute log2 fold-change ≥ 1. Based on these criteria, 63 differentially expressed probe sets corresponding to 49 unique gene symbol entries were identified and carried forward for downstream analyses. Expansion of combined gene annotations (e.g., HBA1/HBA2, TUBA3C/TUBA3D, and LOC100506558/MATN2) yielded 52 individual gene symbols. Of these, 51 genes were successfully represented in the STRING-derived protein–protein interaction network and were therefore included in subsequent network-based analyses and cross-dataset comparisons. Cross-dataset comparison was performed to evaluate the presence and directional consistency of differentially expressed genes in an independent dataset.

### 2.3. Target and Ligand Selection

Candidate targets were selected based on their relevance to key biological processes identified in the differential expression analysis, as well as their established roles in DCM, ferroptosis, fibrosis, and inflammatory signaling [1,6]. Particular emphasis was placed on pathways implicated in oxidative stress, ECM remodeling, and immune-related signaling [2]. Key proteins representing these interconnected processes were included, such as glutathione peroxidase 4 (GPX4) and acyl-CoA synthetase long-chain family member 4 (ACSL4) for ferroptosis [3,4,16] transforming growth factor beta receptor 1 (TGFBR1) and SMAD family member 3 (SMAD3) for fibrotic signaling [6], and signal transducer and activator of transcription 3 (STAT3) and AKT serine/threonine kinase 1 (AKT1) as central mediators linking metabolic stress, inflammation, and tissue remodeling [6,17].

Ligands were selected to represent distinct but complementary pharmacological mechanisms targeting these pathways. Finerenone and pirfenidone were included as representative antifibrotic agents acting on mineralocorticoid receptor–associated and transforming growth factor-β–related signaling pathways [6]. Deferiprone and liproxstatin-1 were selected to probe iron-dependent injury and ferroptosis modulation [16,18]. Bardoxolone methyl was included due to its association with nuclear factor erythroid 2–related factor 2 (NRF2)-mediated antioxidant responses [19], while ruxolitinib was selected to represent inhibition of the Janus kinase/signal transducer and activator of transcription (JAK/STAT) pathway [6].

These selections were intended to systematically represent complementary mechanistic axes within the ferroptosis–fibrosis framework rather than to imply clinical efficacy. Accordingly, the analysis was designed to identify potential multitarget interactions for further investigation rather than to establish therapeutic efficacy. To further contextualize biological relevance, selected targets were cross-checked against publicly available disease–gene association databases (e.g., GeneCards and DisGeNET).

### 2.4. Protein–Protein Interaction (PPI) Network Analysis

PPI network analysis was performed using the STRING database (version 12.0; https://string-db.org/; accessed on 20 May 2026), restricted to Homo sapiens [20]. STRING integrates known and predicted protein–protein associations derived from experimental evidence, computational prediction methods, and curated public databases. The list of differentially expressed genes (DEGs) identified in the present study was uploaded to STRING to construct the interaction network. A high-confidence interaction score threshold (≥0.7) was applied to improve network reliability, and disconnected nodes were hidden to enhance network interpretability. Functional enrichment analysis was performed within STRING using Gene Ontology (GO; Biological Process, Cellular Component, and Molecular Function), KEGG, and Reactome pathway databases. Enriched terms were ranked according to adjusted *p*-values calculated using Fisher’s exact test with Benjamini–Hochberg correction for multiple comparisons. All network and enrichment analyses were intended to characterize biological organization at a systems level rather than to establish causal relationships.

### 2.5. Network Visualization and Topological Analysis

Network visualization and topological analysis were performed using Cytoscape (version 3.10.4; https://cytoscape.org/; accessed on 20 May 2026) [21]. The PPI network generated from STRING was imported into Cytoscape for graphical representation and further analysis. Topological properties, including degree, betweenness centrality, and closeness centrality, were calculated using the Analyze Network function in Cytoscape. Hub genes were identified using the Maximal Clique Centrality (MCC) algorithm implemented in the cytoHubba plugin. The MCC-derived subnetwork was visualized in Cytoscape to illustrate the relationships among the top-ranked hub genes.

### 2.6. Disease Enrichment Analysis

Disease enrichment analysis was performed using the Enrichr platform (https://maayanlab.cloud/Enrichr/; accessed on 20 May 2026) [22] to identify disease-associated annotations for the differentially expressed genes (DEGs) identified in the present study. Gene lists were uploaded to Enrichr and analyzed using disease-related gene set libraries, including DisGeNET and OMIM-based annotations. Enrichment results were ranked based on adjusted *p*-values and combined scores provided by the platform. The most significantly enriched disease terms were selected for further interpretation, with particular attention to cardiometabolic and diabetes-related conditions. This analysis was conducted to facilitate contextualization of the DEG set within disease-relevant biological frameworks rather than to imply direct clinical associations [22].

### 2.7. Exploratory Therapeutic Contextualization

To provide therapeutic contextualization of the identified ferroptosis–fibrosis network, selected candidate compounds with reported relevance to fibrosis, oxidative stress, inflammatory signaling, or iron-dependent cellular injury were reviewed using publicly available pharmacological resources, primarily DrugBank (https://go.drugbank.com/; accessed on 20 May 2026), together with supporting literature sources where appropriate. Candidate compounds were not selected to imply therapeutic efficacy, but rather to contextualize biologically relevant pathways potentially associated with DCM progression and ferroptosis-related stress responses. Mechanistic annotations were summarized descriptively and integrated into the Supplementary Materials as supportive hypothesis-generating information.

### 2.8. Molecular Docking Analysis

Molecular docking was performed to evaluate ligand–target compatibility within fibrosis-, ferroptosis-, inflammatory signaling-, oxidative stress-, and cellular survival–related pathways. Target proteins included TGFBR1 (PDB ID: 1PY5) [23], SMAD3 (PDB ID: 1MJS) [24], STAT3 (PDB ID: 6NJS) [25], AKT1 (PDB ID: 3MV5) [26], GPX4 (PDB ID: 6HKQ) [27], and ACSL4 (AlphaFold model: AF-O60488). ACSL4 was included as a predicted structural model because experimentally resolved ligand-bound structures remain limited; for ACSL4, ligand-binding pocket identification was performed using the CB-Dock2 cavity-detection approach [28]. For GPX4, the selected structure (PDB ID: 6HKQ) contains the catalytically important selenocysteine residue characteristic of glutathione peroxidase family proteins. Because standard AutoDock 4.2.6 atom parameterization may not fully capture the physicochemical properties of selenium-containing residues, GPX4-related docking results should be interpreted with appropriate caution as computational estimates of ligand–target compatibility.

Ligands included finerenone, pirfenidone, deferiprone, liproxstatin-1, bardoxolone methyl, and ruxolitinib. Ligand preparation, receptor preparation, grid definition, and docking simulations were performed using ChemDraw Ultra (v12.0), ChemBio3D Ultra (v13.0), Open Babel (v3.1.1), AutoDockTools (v1.5.6), and AutoDock 4.2.6 employing the Lamarckian Genetic Algorithm (LGA) [29]. For proteins containing co-crystallized ligands, docking grids were centered on ligand-associated binding regions. For each ligand–protein pair, 40 independent docking runs were conducted. Binding poses were evaluated according to predicted binding energy, RMSD, structural compatibility within the binding region, and non-covalent interaction profiles. Conformations with RMSD values ≤ 2 Å were considered reproducible binding modes. Docking outputs were visualized using BIOVIA Discovery Studio Visualizer (2025 Client) and PyMOL (v2.5; Schrödinger, LLC, New York, NY, USA). Predicted binding energies and interaction profiles were interpreted as indicators of structural compatibility and predicted binding tendency. Detailed ligand/receptor preparation procedures, grid parameters, pose-selection criteria, and residue-level interaction analyses are provided in the Supplementary Materials.

### 2.9. Receiver Operating Characteristic (ROC) Analysis

Receiver operating characteristic (ROC) analysis was performed to evaluate the ability of selected candidate hub genes to discriminate between nonfailing control myocardial samples and cardiomyopathy samples in the GSE5406 dataset. The disease group included ischemic and idiopathic cardiomyopathy samples, whereas nonfailing donor myocardial samples were used as controls, consistent with the primary differential expression design. The analysis included candidate genes prioritized from the ECM–centered hub network and fibrosis-related differential expression profile, including COL1A1, COL1A2, COL3A1, LUM, ASPN, THBS1, SERPINE1, PTX3, PDK4, and S100A8.

For each gene, the probe set with the largest absolute log2 fold-change among significant probe sets was used. When the raw expression direction yielded an AUC below 0.50, the score direction was inverted so that all AUC values represented discriminatory performance independent of whether the gene was upregulated or downregulated in cardiomyopathy samples. ROC curves were generated using sample-level normalized expression values. The area under the ROC curve (AUC), 95% CI, sensitivity, specificity, and optimal cutoff values were calculated according to established ROC methodology [30,31]. Optimal cutoff points were determined using the maximum Youden index. The ROC analysis was interpreted as exploratory because of the imbalanced sample distribution between the control and disease groups.

### 2.10. Machine Learning–Based Feature Selection

Machine learning–based feature selection was performed as an exploratory analysis to identify differentially expressed probe-level features with potential discriminatory relevance between nonfailing control and cardiomyopathy samples in the GSE5406 dataset. The analysis was restricted to the 63 differentially expressed probe sets identified in the primary differential expression analysis using an adjusted *p*-value < 0.05 and an absolute log2 fold-change ≥ 1. After excluding features with near-zero variance, three complementary feature-selection approaches were applied: least absolute shrinkage and selection operator (LASSO) logistic regression, random forest classification, and support vector machine–recursive feature elimination (SVM-RFE) [32,33,34]. LASSO logistic regression was used to select variables by applying L1 regularization, random forest analysis was used to rank genes according to feature importance derived from ensemble decision-tree classification, and SVM-RFE was applied to retain features with stronger classification relevance. Probe identifiers were subsequently mapped to gene symbols using the GPL96 platform annotation. Genes selected by all three algorithms were defined as the core machine learning signature, whereas genes selected by at least two algorithms were considered supportive candidate features. Because of the marked imbalance between control and disease samples, these analyses were interpreted as exploratory and hypothesis-generating rather than definitive diagnostic validation. To reduce the risk of overfitting associated with machine-learning analyses, internal validation was performed using stratified class-weighted five-fold cross-validation. Model performance was evaluated by ROC analysis, and the AUC together with corresponding 95% CI was calculated across validation folds. The machine-learning-derived four-gene signature was subsequently subjected to exploratory sample-level external evaluation in GSE263297, as described in Section 2.1.

## 3. Results

### 3.1. Differential Expression Results in GSE5406

Differential expression analysis of the GSE5406 dataset identified a subset of genes exhibiting statistically significant transcriptional alterations between nonfailing myocardial samples and cardiomyopathy samples (ischemic and idiopathic combined). Using predefined thresholds of adjusted *p*-value < 0.05 and absolute log2 fold-change ≥ 1, a total of 63 differentially expressed probe sets corresponding to 49 unique gene symbol entries were retained for downstream analyses, with the top-ranked genes summarized in Table 1. The complete list of differentially expressed probe sets is provided in Supplementary Table S1.

**Figure 1:**
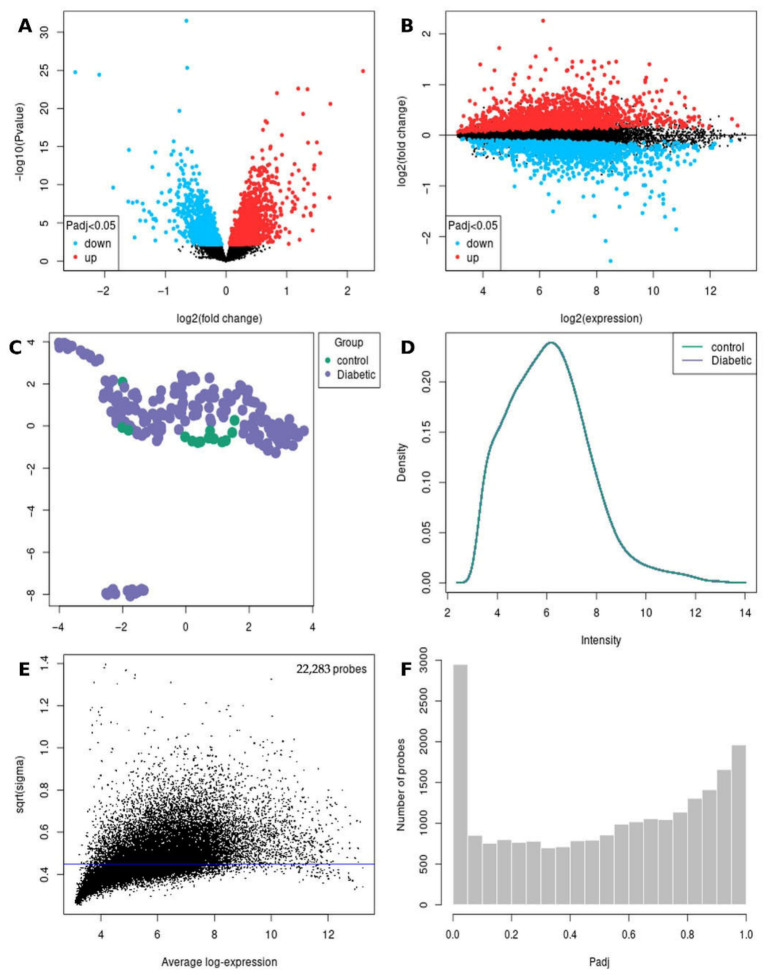
Transcriptomic profiling and differential expression analysis in GSE5406. (**A**) Volcano plot illustrating differentially expressed genes between nonfailing and cardiomyopathy samples. Red and blue points denote significantly upregulated and downregulated genes, respectively (adjusted *p*-value < 0.05). (**B**) Mean–difference (MA) plot showing the relationship between log2 fold-change and average expression levels. (**C**) UMAP plot demonstrating sample distribution and partial separation between control and disease groups. (**D**) Density plot showing comparable global expression distributions across samples, consistent with appropriate normalization. (**E**) Mean–variance trend plot derived from limma analysis, illustrating variance stabilization across expression levels; the blue horizontal line indicates the overall mean residual standard deviation estimated by the limma package. (**F**) Distribution of adjusted *p*-values across all probes, reflecting multiple testing correction and overall statistical behavior.

Visualization of differential expression patterns using a volcano plot demonstrated a broad distribution of transcripts, with a subset exceeding both statistical significance and fold-change thresholds (Figure 1A). While the majority of genes clustered near the center, consistent with relatively modest expression changes, a distinct group of transcripts exhibited more pronounced upregulation and downregulation, consistent with a detectable disease-associated transcriptional signal.

The mean–difference (MA) plot further illustrated the relationship between fold-change and average expression levels, suggesting that differential expression was not restricted to a specific expression range (Figure 1B). Both upregulated and downregulated genes were distributed across a wide span of expression intensities, supporting the robustness of the observed transcriptional differences.

Unsupervised dimensionality reduction using UMAP revealed partial separation between nonfailing and cardiomyopathy samples (Figure 1C). Although a general clustering trend was observed, a degree of overlap between groups persisted, suggesting underlying biological heterogeneity within the cardiomyopathy cohort.

Global expression distributions were highly comparable between groups, as demonstrated by density plots (Figure 1D), consistent with appropriate normalization and absence of major technical bias. This observation was further supported by the mean–variance trend plot (Figure 1E), which demonstrated stable variance across a broad range of expression values, consistent with the assumptions of the limma modeling framework.

Finally, the distribution of adjusted *p*-values showed an expected deviation from the null distribution, with a subset of low *p*-values supporting the presence of true differential expression signals while consistent with appropriate control of multiple testing (Figure 1F).

Taken together, these findings suggest that the GSE5406 dataset captures a detectable yet heterogeneous transcriptional signature associated with cardiomyopathic remodeling. Accordingly, the identified DEG set was considered suitable for subsequent network-based and enrichment analyses.

### 3.2. Cross-Dataset Comparison

To assess the directional consistency of the discovery-cohort transcriptional findings, cross-dataset comparison was performed using the independent GSE263297 dataset. Of the 51 discovery genes evaluated, all 51 were represented in the GSE263297 expression matrix. Directional comparison showed that 20 genes exhibited the same direction of expression change between GSE5406 and GSE263297, corresponding to a directional concordance rate of 39.2% (Table 2). Only one gene showed both same-direction change and an absolute log2 fold-change ≥ 1 in the independent cohort. Because adjusted *p*-values in GSE263297 did not support statistically significant replication of the discovery findings, this analysis was interpreted as directional comparison rather than robust external validation.

For the independent dataset, log2 fold-change values were recalculated using normalized expression data by comparing ICM-DM samples (*n* = 7) with donor controls (*n* = 7). Statistical comparisons were performed using Welch’s *t*-test, and *p*-values were adjusted using the Benjamini–Hochberg false discovery rate method. Among the concordant genes, MAFF demonstrated the largest effect size in GSE263297 (log2FC = 1.24), whereas the remaining 19 genes showed attenuated expression differences in the independent cohort, with absolute log2 fold-change values below 1. Although none of the concordant genes remained statistically significant after multiple-testing correction in GSE263297, the observed directional agreement between datasets suggests limited cross-cohort consistency of the transcriptional trends identified in GSE5406. The reduced effect sizes and lack of statistical significance in the independent cohort may reflect differences in cohort composition, sample size, disease characteristics, and transcriptomic platform (microarray versus RNA sequencing).

### 3.3. PPI Network and Functional Enrichment Analysis

**Figure 2:**
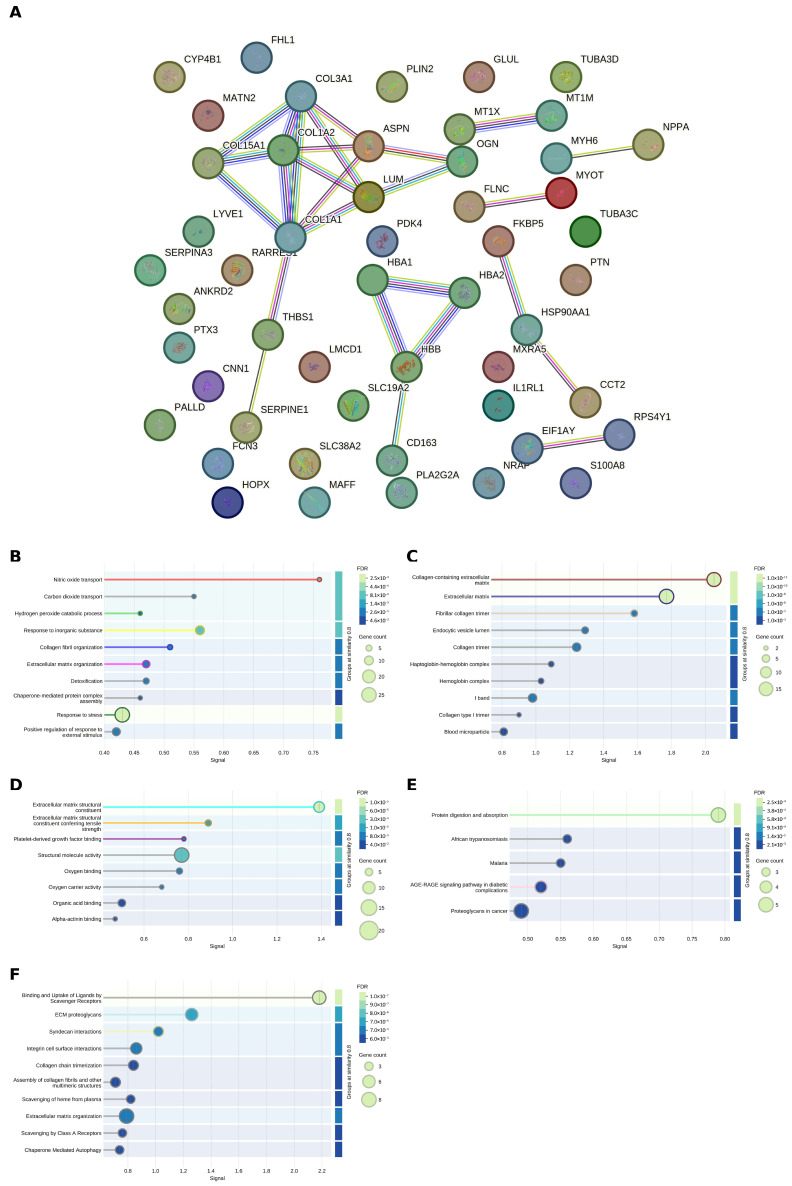
STRING-based protein–protein interaction network and functional enrichment analysis of the differentially expressed gene set. (**A**) Protein–protein interaction (PPI) network generated using the STRING database for the DEG-derived input list. Nodes represent proteins, and edges represent known or predicted functional associations. (**B**) Gene Ontology Biological Process (GO-BP) enrichment analysis. (**C**) Gene Ontology Cellular Component (GO-CC) enrichment analysis. (**D**) Gene Ontology Molecular Function (GO-MF) enrichment analysis. (**E**) Kyoto Encyclopedia of Genes and Genomes (KEGG) pathway enrichment analysis. (**F**) Reactome pathway enrichment analysis. In the enrichment plots, bubble size represents the number of genes associated with each term, and color intensity represents false discovery rate (FDR)-adjusted significance.

The PPI network constructed from the DEG-derived input list comprised 51 nodes and 26 edges, with a PPI enrichment *p*-value of 8.78 × 10^−12^, suggesting that the observed interactions significantly exceeded random expectation and indicating non-random functional connectivity within the network (Figure 2A). Topological inspection revealed a limited number of moderately connected nodes, with COL1A1 (degree = 6), and COL1A2 and COL3A1 (degree = 5) emerging as the most connected elements. In contrast, a substantial proportion of nodes exhibited low or zero degree, consistent with a partially fragmented network structure (Figure 2A). This pattern may reflect the coexistence of a central ECM-related module alongside more isolated functional components.

Gene Ontology (GO) enrichment analysis demonstrated a consistent pattern centered on ECM organization and stress-related processes (Figure 2B–D, Table 3). Within the Biological Process category, enrichment was observed for response to stress (25 genes; FDR = 0.0025), response to inorganic substance (10 genes; FDR = 0.0071), and nitric oxide transport (3 genes; FDR = 0.0071) (Figure 2B, Table 3). These broadly defined processes may suggest activation of adaptive and redox-related responses rather than a single dominant signaling axis. In the Cellular Component category, the most prominent enrichments were collagen-containing ECM (16 genes; FDR = 1.09 × 10^−11^) and ECM (17 genes; FDR = 3.12 × 10^−11^) (Figure 2C, Table 3), suggesting that a substantial fraction of the network localizes to extracellular structural compartments, consistent with the central positioning of collagen-related nodes. Within the Molecular Function category, enrichment was observed for ECM structural constituent (8 genes; FDR = 1.20 × 10^−5^) and structural molecule activity (13 genes; FDR = 0.00017) (Figure 2D, Table 3). This profile may reflect a predominance of structural and scaffold-associated proteins rather than enzymatic or receptor-driven activities.

Pathway-level analysis further supported these observations (Figure 2E,F, Table 3). In KEGG, enrichment was identified for protein digestion and absorption (FDR = 0.0025) and the AGE–RAGE signaling pathway in diabetic complications (FDR = 0.0216) (Figure 2E, Table 3). Given the gene composition—particularly collagen isoforms—the former likely reflects structural protein turnover rather than gastrointestinal physiology. In Reactome, enriched pathways included ECM proteoglycans (FDR = 7.53 × 10^−5^), ECM organization (FDR = 8.6 × 10^−4^), integrin cell surface interactions, and collagen chain trimerization (Figure 2F, Table 3). Additionally, binding and uptake of ligands by scavenger receptors demonstrated strong enrichment (FDR = 1.04 × 10^−7^), which may suggest involvement of macrophage-related or clearance-associated processes.

Taken together, these findings suggest that the network is organized around ECM structure and remodeling, stress-responsive processes, and receptor-mediated ligand handling pathways. However, given the relatively small network size, partial fragmentation, and heterogeneity of enriched terms, these results should be interpreted as exploratory and hypothesis-generating, rather than definitive evidence of a unified mechanistic pathway.

### 3.4. Cytoscape-Based Network Analysis

Network topology analysis performed in Cytoscape demonstrated heterogeneous node connectivity within the MCC-derived subnetwork. Degree, betweenness centrality, and closeness centrality values are summarized (Table 4).

Among the analyzed nodes, COL1A1 exhibited the highest degree and betweenness centrality, consistent with a prominent position in maintaining network connectivity within this subnetwork. COL1A2 and COL3A1 also showed relatively high centrality values, whereas several nodes, including HSP90AA1 and HBB, displayed minimal or absent connectivity.

In contrast, ranking based on MCC scores revealed a partially distinct pattern of hub gene prioritization (Table 5).

**Figure 3:**
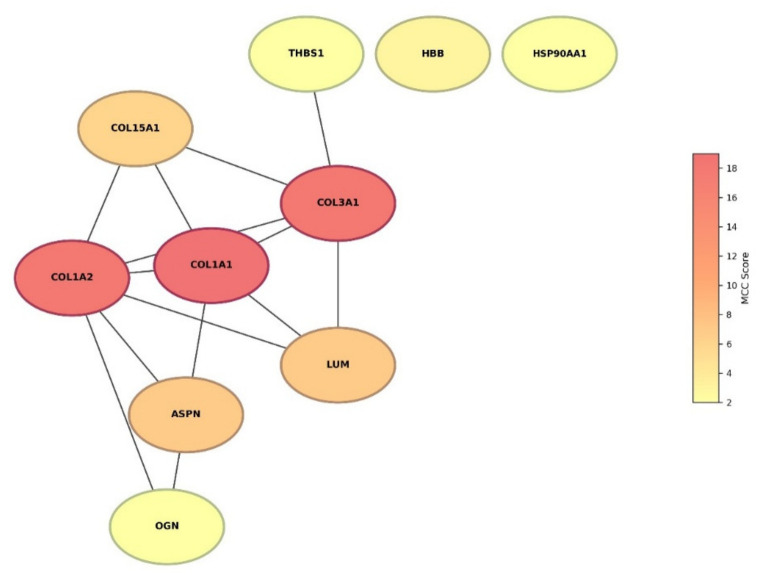
PPI subnetwork of hub genes identified using the MCC algorithm in the cytoHubba plugin of Cytoscape. Node color represents MCC scores, with red corresponding to higher centrality and yellow to lower centrality.

Specifically, *COL15A1*, *ASPN*, and *LUM* demonstrated the highest MCC scores, while COL1A1 and other collagen-related genes showed comparatively lower MCC values. Notably, some nodes with low or absent degree centrality were also associated with low MCC scores. Visualization of the MCC-derived subnetwork illustrates a structured cluster primarily composed of ECM–related genes (Figure 3).

The observed differences between degree-based and MCC-based rankings suggest that these centrality measures capture distinct topological features of the network. These findings should therefore be interpreted in the context of a topologically limited subnetwork.

### 3.5. Disease Enrichment Results

Disease enrichment analysis was performed using the DisGeNET and OMIM Disease libraries in the Enrichr platform to explore potential disease-associated patterns within the differentially expressed gene set (Table 6).

**Figure 4:**
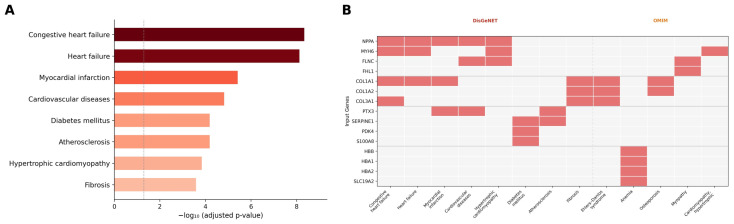
Disease enrichment analysis of differentially expressed genes using DisGeNET and OMIM databases. (**A**) Bar plot showing the top enriched disease terms identified from the DisGeNET database, ranked according to adjusted *p*-values. The analysis highlights associations with cardiovascular conditions (including congestive heart failure and myocardial infarction), connective tissue disorders (such as Ehlers–Danlos syndrome), and hemoglobin-related traits. Darker colors indicate higher statistical significance (higher −log10 adjusted *p*-values), whereas lighter colors indicate lower statistical significance. (**B**) Clustergram illustrating the relationship between selected enriched disease terms and input genes. Hemoglobin-related genes (*HBB*, *HBA1*, *HBA2*) cluster with erythroid and thalassemia-associated terms, while ECM–related genes (*COL1A1*, *COL1A2*, *COL3A1*, *LUM*) show associations with connective tissue–related conditions. These patterns suggest that the identified gene set may be linked to both erythroid and ECM–associated disease processes.

DisGeNET analysis demonstrated a strong overrepresentation of cardiovascular-related conditions, including congestive heart failure and heart failure, which showed the most significant adjusted *p*-values (Figure 4A). Additional enriched terms included myocardial infarction, hypertrophic cardiomyopathy, and broader cardiovascular disease categories. These enrichments were supported by genes involved in cardiac structure and function, such as *NPPA*, *MYH6*, and *FLNC*.

In addition to cardiac-related terms, several cardiometabolic conditions, including diabetes mellitus and atherosclerosis, were also enriched. These findings suggest overlap between the identified transcriptional profile and systemic processes commonly associated with cardiac remodeling.

OMIM Disease analysis provided complementary results, highlighting enrichment of connective tissue and structural disorders, including Ehlers–Danlos syndrome and osteoporosis, largely driven by collagen-related genes (*COL1A1*, *COL1A2*, *COL3A1*). Enrichment of anemia-related terms was also observed, reflecting the presence of hemoglobin gene family members (*HBA1*, *HBA2*, *HBB*).

The gene–disease association structure further revealed distinct clustering patterns (Figure 4B), where hemoglobin-related genes grouped with erythroid and thalassemia-associated terms, while ECM–related genes showed associations with connective tissue–related conditions.

These disease associations were broad and partially overlapping, and therefore were interpreted cautiously. Overall, disease enrichment analysis provides contextual support for a transcriptional profile centered on cardiac remodeling, ECM organization, and cardiometabolic conditions (Figure 4). Additional OMIM-based visualizations are provided in Supplementary Figure S1.

### 3.6. Pathway Enrichment Results

Pathway enrichment analysis was performed using the KEGG 2026 and Reactome 2024 databases through Enrichr (Table 7). These pathway enrichment results are independent of the STRING functional enrichment analysis presented in Table 3; therefore, adjusted *p*-values may differ between the two analyses.

**Figure 5:**
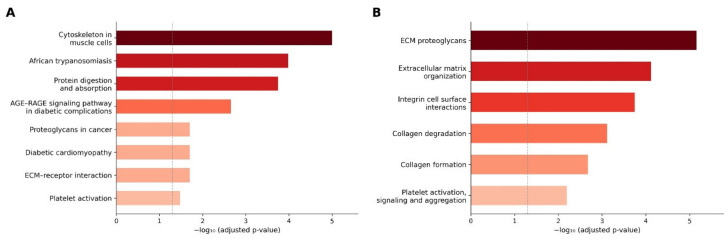
Pathway enrichment analysis based on KEGG 2026 and Reactome 2024 databases. (**A**) KEGG pathway enrichment results showing the top significantly enriched pathways, including cytoskeleton organization in muscle cells, ECM–receptor interaction, AGE–RAGE signaling pathway in diabetic complications, and the KEGG pathway Proteoglycans in cancer. (**B**) Reactome pathway enrichment analysis demonstrating enrichment of ECM-related processes, including ECM proteoglycans, ECM organization, and collagen-related pathways. Across both databases, enrichment patterns consistently point toward processes involving ECM organization, proteoglycan-associated pathways, and cytoskeletal structure, accompanied by signals related to inflammatory and metabolic processes. These findings provide a systems-level context for the observed gene expression changes. Darker colors indicate higher statistical significance (higher −log10 adjusted *p*-values), whereas lighter colors indicate lower statistical significance.

KEGG analysis identified several pathways related to structural organization and metabolic processes (Figure 5A). Among the most significantly enriched pathways were cytoskeleton organization in muscle cells and protein digestion and absorption, the latter likely reflecting the overrepresentation of collagen-related genes. Additional pathways included AGE–RAGE signaling in diabetic complications, ECM–receptor interaction, and DCM, suggesting involvement of ECM remodeling and cardiometabolic processes.

Reactome analysis further emphasized ECM-related pathways (Figure 5B), including ECM organization, ECM proteoglycans, collagen formation, and integrin-mediated interactions. These findings are consistent with the prominent representation of collagen and matrix-associated genes within the dataset.

Additional pathways related to platelet activation and signaling were also identified, suggesting a potential link to vascular or hemostatic processes. However, similar to the disease enrichment results, these pathways were relatively broad and partially overlapping.

Overall, pathway enrichment analysis is consistent with a network organization centered on ECM remodeling, structural integrity, and stress-related signaling processes (Figure 5), while remaining descriptive in nature. Detailed pathway–gene association patterns are shown in Supplementary Figures S2 and S3.

### 3.7. Exploratory Therapeutic Contextualization of Candidate Compounds

To provide additional mechanistic contextualization of the identified ferroptosis–fibrosis network, selected candidate compounds associated with fibrosis, oxidative stress, inflammatory signaling, and iron-dependent cellular injury pathways were reviewed using publicly available pharmacological resources and supporting literature sources. As summarized in Supplementary Table S2, the selected compounds demonstrated mechanistic relevance to several biologically interconnected processes implicated in DCM, including ECM remodeling, inflammatory signaling, oxidative stress responses, and ferroptosis-associated cellular injury. These observations were not intended to imply therapeutic efficacy, but rather to provide pathway-level contextualization supporting the biological rationale underlying the subsequent molecular docking analyses.

### 3.8. Docking Results

**Figure 6:**
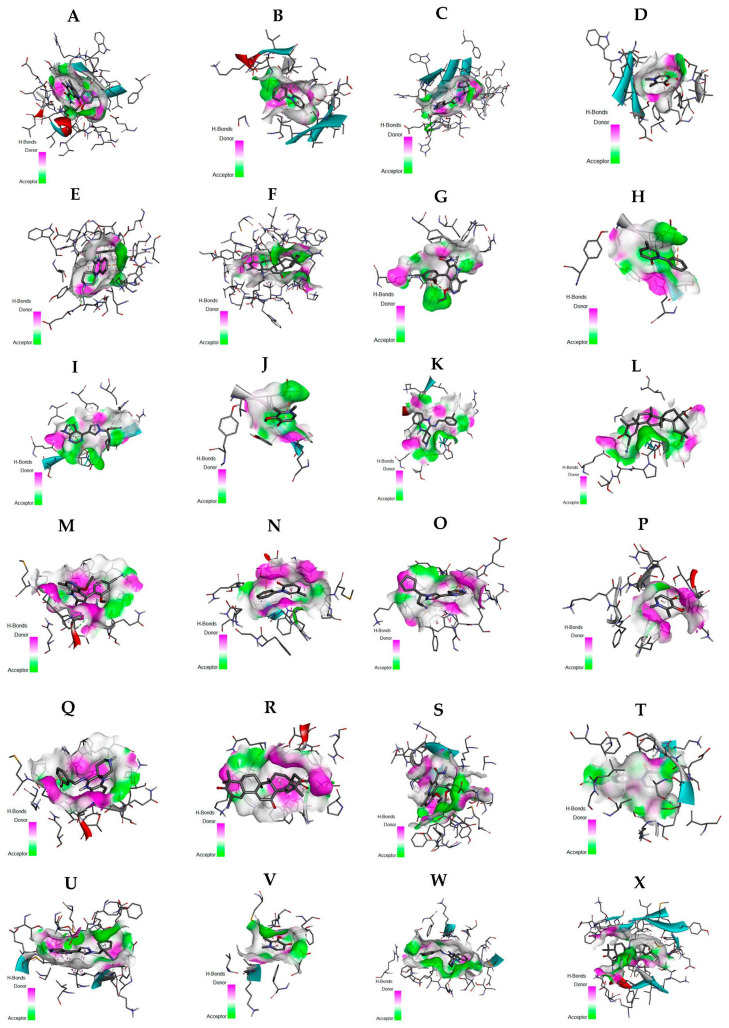
3D docking conformations of candidate compounds within the predicted binding regions of TGFBR1, STAT3, GPX4, and AKT1. (**A**) Finerenone–TGFBR1, (**B**) Pirfenidone–TGFBR1, (**C**) Ruxolitinib–TGFBR1, (**D**) Deferiprone–TGFBR1, (**E**) Liproxstatin-1–TGFBR1, (**F**) Bardoxolone methyl–TGFBR1, (**G**) Finerenone–STAT3, (**H**) Pirfenidone–STAT3, (**I**) Ruxolitinib–STAT3, (**J**) Deferiprone–STAT3, (**K**) Liproxstatin-1–STAT3, (**L**) Bardoxolone methyl–STAT3, (**M**) Finerenone–GPX4, (**N**) Pirfenidone–GPX4, (**O**) Ruxolitinib–GPX4, (**P**) Deferiprone–GPX4, (**Q**) Liproxstatin-1–GPX4, (**R**) Bardoxolone methyl–GPX4, (**S**) Finerenone–AKT1, (**T**) Pirfenidone–AKT1, (**U**) Ruxolitinib–AKT1, (**V**) Deferiprone–AKT1, (**W**) Liproxstatin-1–AKT1, and (**X**) Bardoxolone methyl–AKT1. Representative low-energy docking poses are shown within the predicted binding regions of the respective target proteins. Ligands are displayed in stick representation, whereas surrounding protein structures are visualized using backbone, residue-level, and semi-transparent molecular surface representations to facilitate inspection of ligand orientation and local binding-site geometry. Docking conformations were generated using AutoDock 4.2.6 and visualized using PyMOL.

**Figure 7:**
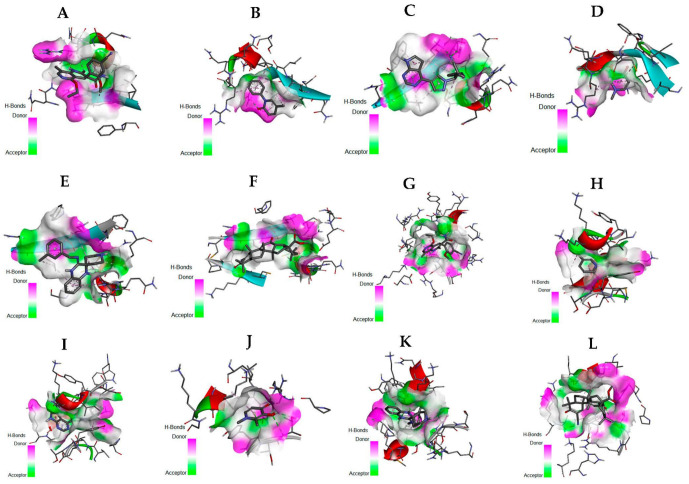
3D docking conformations of candidate compounds within the predicted binding regions of SMAD3 and ACSL4. (**A**) Finerenone–SMAD3, (**B**) Pirfenidone–SMAD3, (**C**) Ruxolitinib–SMAD3, (**D**) Deferiprone–SMAD3, (**E**) Liproxstatin-1–SMAD3, (**F**) Bardoxolone methyl–SMAD3, (**G**) Finerenone–ACSL4, (**H**) Pirfenidone–ACSL4, (**I**) Ruxolitinib–ACSL4, (**J**) Deferiprone–ACSL4, (**K**) Liproxstatin-1–ACSL4, and (**L**) Bardoxolone methyl–ACSL4. Representative low-energy docking poses are shown within the predicted binding regions of the respective target proteins. Ligands are displayed in stick representation, whereas surrounding protein structures are visualized using backbone, residue-level, and semi-transparent molecular surface representations to facilitate inspection of ligand orientation and local binding-site geometry. Docking conformations were generated using AutoDock 4.2.6 and visualized using PyMOL.

Molecular docking analyses were performed to provide structural context for selected ligand–target combinations within fibrosis-, ferroptosis-, inflammatory signaling-, oxidative stress-, and cellular survival–related pathways. Representative three-dimensional docking conformations are presented in Figure 6 and Figure 7. Detailed docking parameters and grid definitions are provided in Supplementary Table S3.

The chemical structures of all candidate compounds included in the docking analyses are provided in Supplementary Figure S4. Detailed residue-level interaction profiles, including conventional hydrogen bonds, π-cation/π-anion interactions, alkyl/π-alkyl contacts, and π-sigma interactions, are summarized in Supplementary Table S4. Two-dimensional protein–ligand interaction diagrams for all analyzed protein–ligand complexes are provided in Supplementary Figures S5–S8.

Across the analyzed ligand–target combinations, predicted binding energy values varied according to both compound and target protein. For TGFBR1, the numerically lowest predicted binding energy values were recorded for ruxolitinib (−12.08 kcal/mol), liproxstatin-1 (−11.39 kcal/mol), and finerenone (−10.40 kcal/mol). For STAT3, the lowest numerical values were recorded for liproxstatin-1 (−9.03 kcal/mol), bardoxolone methyl (−8.69 kcal/mol), ruxolitinib (−8.59 kcal/mol), and finerenone (−8.49 kcal/mol). For GPX4, ruxolitinib (−8.06 kcal/mol), liproxstatin-1 (−7.65 kcal/mol), and finerenone (−7.19 kcal/mol) showed the lowest numerical binding energy values within this target group. For AKT1, lower numerical binding energy values were recorded for ruxolitinib (−11.72 kcal/mol), liproxstatin-1 (−10.10 kcal/mol), bardoxolone methyl (−8.75 kcal/mol), and finerenone (−8.60 kcal/mol). In the SMAD3 docking set, liproxstatin-1 (−8.55 kcal/mol), ruxolitinib (−7.84 kcal/mol), and finerenone (−7.18 kcal/mol) yielded the lowest numerical values. For ACSL4, liproxstatin-1 (−10.86 kcal/mol), ruxolitinib (−10.75 kcal/mol), and finerenone (−10.20 kcal/mol) had the lowest predicted binding energy values among the evaluated compounds. Notably, although ruxolitinib yielded the numerically lowest predicted binding energy value for TGFBR1, this observation should be interpreted cautiously because ruxolitinib is a clinically established JAK1/JAK2 inhibitor rather than a known TGFBR1-directed ligand. Therefore, the observed docking score may reflect structural compatibility within the modeled binding region rather than biologically relevant target selectivity.

The retained docking poses were associated with RMSD values within the predefined acceptable range, and residue-level interaction patterns varied across ligand–target pairs. These interaction profiles are presented in detail in Supplementary Table S4 and were used only to describe the docking conformations retained for structural interpretation.

Overall, the docking results are presented as exploratory computational observations intended to provide structural context for the investigated ligand–target combinations. Predicted binding energies and interaction patterns were not interpreted as evidence of biological activity, target inhibition, pathway modulation, or therapeutic efficacy.

### 3.9. Exploratory ROC Analysis of Candidate Hub Genes

**Figure 8:**
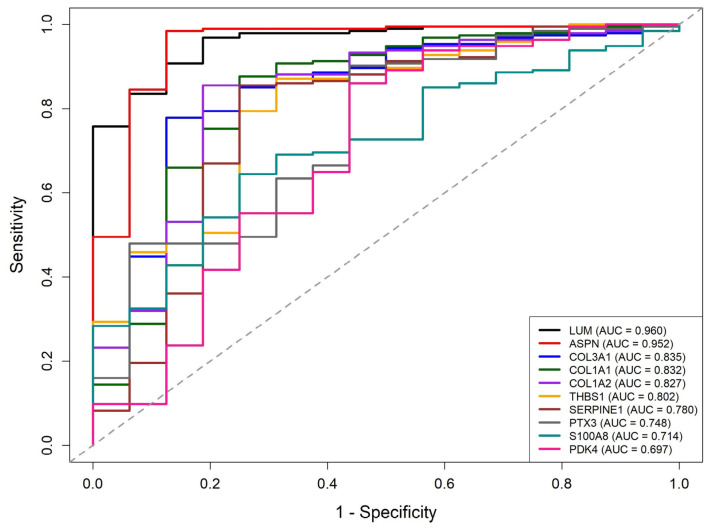
Receiver operating characteristic (ROC) curves of ten candidate hub genes in the GSE5406 dataset. ROC curves were generated using normalized gene expression values to evaluate the discriminatory performance of candidate genes between cardiomyopathy and nonfailing myocardial samples. *LUM* and *ASPN* showed the highest AUC values among the evaluated genes. The dashed diagonal line represents the line of no discrimination (AUC = 0.5), corresponding to the performance expected by random classification.

ROC analysis was performed to evaluate the discriminatory performance of ten candidate hub genes between nonfailing control myocardial samples and cardiomyopathy samples in the GSE5406 dataset. Among the evaluated genes, LUM demonstrated the highest diagnostic performance (AUC = 0.960, 95% CI: 0.923–0.997), followed by *ASPN* (AUC = 0.952, 95% CI: 0.889–1.000), *COL3A1* (AUC = 0.835, 95% CI: 0.715–0.954), *COL1A1* (AUC = 0.832, 95% CI: 0.703–0.960), *COL1A2* (AUC = 0.827, 95% CI: 0.705–0.949), *THBS1* (AUC = 0.802, 95% CI: 0.686–0.919), *SERPINE1* (AUC = 0.780, 95% CI: 0.632–0.929), *PTX3* (AUC = 0.748, 95% CI: 0.615–0.881), *S100A8* (AUC = 0.714, 95% CI: 0.598–0.830), and *PDK4* (AUC = 0.697, 95% CI: 0.532–0.861) (Figure 8, Table 8).

For ROC analysis, score direction was inverted only for *THBS1*, *SERPINE1*, *PTX3*, *S100A8*, and *PDK4* when the raw expression direction yielded an AUC below 0.50. No inversion was applied for *LUM*, *ASPN*, *COL3A1*, *COL1A1*, or *COL1A2.* logFC values reflect the control − disease direction according to GEO2R group assignment.

*LUM* and *ASPN* exhibited the highest AUC values among the evaluated candidate hub genes, followed by *COL3A1*, *COL1A1*, *COL1A2*, and *THBS1*. *SERPINE1* and *PTX3* showed moderate discriminatory performance, whereas S100A8 and PDK4 showed lower AUC values. Overall, these findings may reflect the contribution of ECM-associated and fibrosis-related genes to sample-level separation between cardiomyopathy and nonfailing myocardial samples in the discovery cohort. However, because ROC analysis was performed within the same dataset used for differential-expression discovery and because of the unequal sample distribution between groups, these results should be interpreted as exploratory and hypothesis-generating rather than evidence of definitive diagnostic performance.

### 3.10. Machine Learning–Based Identification of Candidate Diagnostic Signatures

LASSO regression identified 9 candidate genes, whereas random forest and SVM-RFE selected 18 and 34 genes, respectively (Table 9).

**Figure 9:**
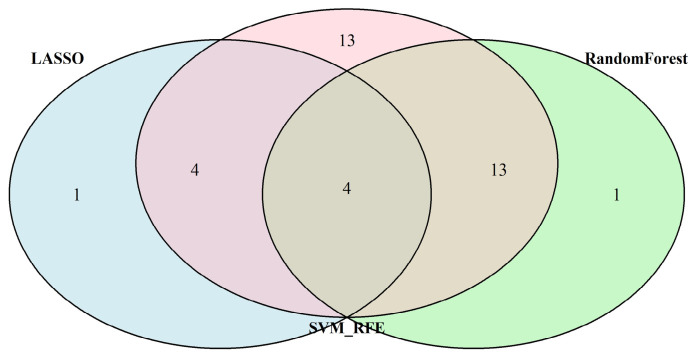
Venn diagram showing the overlap of candidate genes identified by least absolute shrinkage and selection operator (LASSO) regression, random forest classification, and support vector machine–recursive feature elimination (SVM-RFE) in the GSE5406 dataset. Four genes (*FCN3*, *HOPX*, *CNN1*, and *GLUL*) were consistently selected by all three machine learning algorithms and were therefore defined as the core machine learning signature. Additional genes were shared between two algorithms, further supporting convergent machine-learning prioritization of candidate cardiomyopathy-associated features. Feature-selection results should be interpreted as exploratory and hypothesis-generating. Numbers within each region indicate the number of genes uniquely or jointly identified by the corresponding machine learning methods.

Comparison of the three feature-selection approaches demonstrated partial overlap among genes associated with ECM remodeling, myocardial structure, inflammation, and metabolic regulation. The overlap analysis demonstrated convergence of machine-learning prioritization despite methodological differences among the algorithms (Figure 9). Because feature selection was performed within the same discovery cohort and under marked class imbalance, these findings should be regarded as exploratory and hypothesis-generating.

**Figure 10:**
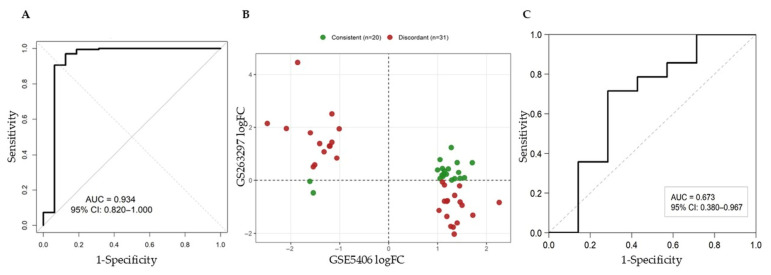
Exploratory evaluation analyses of the machine-learning–derived gene signature. (**A**) Internal validation of the machine-learning framework using stratified class-weighted five-fold cross-validation. To address potential overfitting and class imbalance within the discovery cohort, internal validation was performed using out-of-fold evaluation. The model achieved a cross-validated AUC of 0.934 (95% CI: 0.820–1.000). Class weighting was applied to account for the imbalance between cardiomyopathy samples (*n* = 194) and nonfailing control samples (*n* = 16). (**B**) Cross-cohort comparison of differential-expression patterns between the GSE5406 discovery cohort and the independent GSE263297 cohort. Each point represents a gene identified in the GSE5406 discovery analysis and present in the GSE263297 dataset. The x-axis indicates logFC values in GSE5406, whereas the y-axis indicates logFC values in GSE263297. Dashed lines denote zero logFC. Green points indicate genes with consistent direction of expression change across cohorts, whereas red points indicate discordant genes. Among the 51 genes evaluated in both cohorts, 20 exhibited the same direction of expression change, while one gene retained both directional consistency and an absolute validation logFC ≥ 1. These findings provide exploratory cross-cohort observations regarding the identified transcriptional patterns but should not be interpreted as formal external validation of the machine-learning signature. (**C**) Receiver operating characteristic (ROC) analysis of the machine-learning–derived four-gene signature in the independent GSE263297 cohort. A composite score based on *FCN3*, *HOPX*, *CNN1*, and *GLUL* expression was evaluated using normalized log2-transformed RNA-sequencing data from ICM-DM and donor myocardial samples. The ROC curve provides an exploratory sample-level evaluation of the four-gene signature in an independent cohort. The corresponding AUC and 95% CI are displayed within the figure. The dashed diagonal line represents the line of no discrimination (AUC = 0.5), corresponding to random classification.

To address the risk of overfitting and class imbalance, the machine-learning framework was further evaluated using stratified class-weighted five-fold cross-validation. The internally validated model achieved an AUC of 0.934 (95% CI: 0.820–1.000), with class weighting applied to account for the imbalance between cardiomyopathy samples and nonfailing controls (Figure 10A, Table 10(A)). Cross-cohort comparison between GSE5406 and GSE263297 showed that all 51 discovery genes were present in the independent cohort, with 20 genes showing the same direction of expression change and one gene retaining both directional consistency and |logFC| ≥ 1 (Figure 10B, Table 10(B)). Sample-level external evaluation of the *FCN3*–*HOPX*–*CNN1*–*GLUL* four-gene signature in GSE263297 yielded an AUC of 0.673 (95% CI: 0.380–0.967) (Figure 10C, Table 10(C)). Among individual genes, *FCN3* showed the highest AUC (0.806), whereas *HOPX*, *GLUL*, and *CNN1* showed lower AUC values. Together, these analyses provide exploratory internal and independent-cohort observations for the machine-learning–derived signature, while remaining insufficient to establish clinical diagnostic utility.

Internal validation was performed using stratified class-weighted five-fold cross-validation. Cross-cohort comparison assessed directional concordance of differential-expression patterns between GSE5406 and GSE263297. External sample-level evaluation was conducted using normalized log2-transformed RNA-sequencing expression data from the independent GSE263297 cohort.

## 4. Discussion

This study was designed to explore whether transcriptional alterations identified in cardiomyopathic myocardial tissue may exhibit an underlying network organization reflecting ECM remodeling and stress-related biological processes.

At the level of individual differentially expressed genes, several transcripts identified in the present analysis are consistent with previously described structural and stress-related alterations in cardiomyopathic remodeling. In particular, the prominent representation of ECM–associated genes, including *COL1A1*, *COL1A2*, *COL3A1*, *LUM*, and *ASPN*, may reflect ongoing matrix remodeling processes. Similarly, the presence of stress- and inflammation-related genes, such as *SERPINE1*, *PTX3*, and *S100A8*, may indicate broader adaptive or injury-related responses.

However, differential expression alone does not establish functional involvement, and the observed gene-level alterations may reflect downstream or compensatory responses rather than primary disease drivers. In addition, the partial concordance observed in cross-dataset comparisons limits the generalizability of individual gene-level findings. Accordingly, interpretation at the single-gene level should be approached with caution and considered within a broader network and pathway context.

The present study identified a transcriptional signature associated with cardiomyopathic remodeling and explored its potential biological organization using network-based and enrichment analyses. Although differential expression analysis revealed a detectable set of altered transcripts, cross-dataset comparison demonstrated only partial preservation of expression patterns, suggesting that the observed signal is likely context-dependent and biologically heterogeneous rather than universally conserved.

From a network perspective, the PPI analysis suggested a relatively sparse and partially fragmented structure, with a limited number of moderately connected nodes. Notably, collagen-related proteins (COL1A1, COL1A2, and COL3A1) emerged as the most connected elements, suggesting that ECM–associated components may represent a central structural axis within the network. The absence of a densely interconnected core indicates that the transcriptional alterations identified here do not converge on a single dominant pathway but instead reflect multiple coexisting biological processes.

Functional enrichment analysis further supported this interpretation. Gene Ontology results demonstrated enrichment in ECM organization and structural components, particularly within the Cellular Component and Molecular Function categories. These findings are consistent with the central positioning of collagen isoforms in the PPI network and may reflect remodeling of extracellular architecture rather than activation of a discrete signaling cascade.

At the same time, enrichment of broadly defined Biological Process terms, such as response to stress and response to inorganic substance, suggests activation of adaptive and redox-related processes. Given the nonspecific nature of these categories, these observations should be interpreted cautiously and may represent generalized stress responses rather than pathway-specific regulation.

Pathway-level analyses provided additional context but should be interpreted with similar caution. The enrichment of protein digestion and absorption in KEGG, in the presence of collagen-rich gene input, likely reflects structural protein turnover rather than gastrointestinal physiology. Similarly, the identification of the AGE–RAGE signaling pathway may be influenced by the inclusion of genes associated with ECM remodeling and diabetic complications, rather than reflecting direct pathway activation.

Interestingly, recent evidence suggests that ferroptosis may represent a mechanistic link between metabolic stress, oxidative injury, and myocardial remodeling in DCM. Experimental and translational studies have implicated ferroptosis-related pathways in the development of myocardial fibrosis, ECM accumulation, and adverse cardiac remodeling, providing biological context of ferroptosis-associated signals identified in the present analysis [35,36,37].

Reactome analysis highlighted ECM organization, ECM proteoglycans, and integrin-mediated interactions, further reinforcing the concept that extracellular structural remodeling represents a prominent feature of the observed network. The enrichment of scavenger receptor–related pathways may additionally suggest involvement of clearance-associated or macrophage-related processes, although this interpretation remains speculative.

In addition to functional enrichment, disease-oriented annotation provided complementary contextual information. Disease enrichment analysis using DisGeNET and OMIM libraries demonstrated overrepresentation of cardiovascular conditions, including heart failure, myocardial infarction, and cardiomyopathy-related terms. These associations were supported by genes involved in cardiac structure and contractile function, such as *NPPA*, *MYH6*, *FLNC*, and *FHL1*.

Furthermore, enrichment of connective tissue and ECM–related disorders, including Ehlers–Danlos syndrome, was observed, reflecting the prominence of collagen genes (*COL1A1*, *COL1A2*, *COL3A1*) within the dataset. Metabolic and cardiometabolic pathways, including AGE–RAGE signaling and DCM, were also represented, suggesting potential overlap between extracellular remodeling processes and systemic metabolic stress. However, enrichment of hemoglobin-related disease categories was also detected, likely driven by the inclusion of *HBA* and *HBB* gene family members, and should therefore be interpreted cautiously.

Taken together, these findings suggest that the observed transcriptional profile is organized around ECM remodeling, structural integrity, and cardiometabolic stress-related processes. Accordingly, the fibrosis component of the proposed ferroptosis–fibrosis axis is more directly supported by the transcriptomic findings, whereas the ferroptosis component should be interpreted as an integrative, hypothesis-generating biological framework informed by prior mechanistic knowledge and exploratory docking analysis rather than as a directly demonstrated transcriptomic mechanism.

Recent evidence has increasingly highlighted potential mechanistic links between ferroptosis, myocardial fibrosis, oxidative stress, and cardiometabolic remodeling, suggesting that ferroptosis-associated pathways may contribute to ECM remodeling and adverse cardiac remodeling in cardiovascular disease states, including cardiomyopathy [16,36,37,38].

ROC and machine learning analyses provided additional contextual information regarding the identified remodeling signature. Among the evaluated candidate hub genes, LUM and ASPN exhibited the highest discriminatory performance between cardiomyopathy and nonfailing myocardial samples. *COL3A1*, *COL1A1*, *COL1A2*, and *THBS1* also demonstrated relatively strong classification capacity, whereas *SERPINE1*, *PTX3*, *S100A8*, and *PDK4* showed moderate discriminatory performance. Notably, these genes are closely linked to ECM organization and myocardial structural remodeling, reinforcing the central role of matrix-related processes identified through differential expression, network, and enrichment analyses.

Machine learning–based feature selection further refined candidate gene prioritization from a complementary analytical perspective. Despite methodological differences among LASSO regression, random forest classification, and *SVM-RFE*, *FCN3*, *HOPX*, *CNN1*, and *GLUL* were consistently identified across all three algorithms, constituting the core machine learning signature. The convergence of independent feature-selection approaches on a limited subset of genes suggests that these candidates may represent relatively stable components of the observed cardiomyopathy-associated transcriptional landscape. In particular, the identification of *LUM* by ROC analysis and by two of three machine learning algorithms provides additional context for its potential relevance within ECM-associated remodeling processes. *HOPX*, a transcriptional regulator implicated in cardiac development and stress-responsive remodeling, and *CNN1*, a cytoskeleton-associated marker linked to smooth muscle-like contractile and remodeling phenotypes, are consistent with the fibrosis-dominant and structural remodeling signature observed in the present analysis.

Nevertheless, these findings should be interpreted within the exploratory framework of the present study. ROC analysis and machine-learning–based feature selection were performed within the GSE5406 discovery cohort, and additional validation analyses were conducted to reduce the risk of overfitting. Stratified class-weighted five-fold cross-validation demonstrated preserved internal discriminatory performance, while exploratory sample-level external evaluation of the *FCN3*–*HOPX*–*CNN1*–*GLUL* signature in the independent GSE263297 cohort yielded an AUC of 0.673 (95% CI: 0.380–0.967). Nevertheless, these findings should still be interpreted cautiously because the external cohort was limited in size and differed from the discovery cohort in disease context, sample composition, and transcriptomic platform. Therefore, the results inform exploratory candidate-gene prioritization rather than definitive diagnostic validation or clinical utility.

Within this framework, mechanistic contextualization of selected candidate compounds further suggested potential biological convergence between ECM remodeling, oxidative stress, inflammatory signaling, and ferroptosis-associated cellular injury pathways. Although these observations do not imply therapeutic efficacy, the integration of compounds associated with mineralocorticoid signaling, iron handling, oxidative stress modulation, and cytokine-related pathways provided an additional systems-level perspective supporting the biological plausibility of the identified ferroptosis–fibrosis network.

In addition, molecular docking analyses provided a complementary structural perspective by examining selected ligand–target combinations within fibrosis-, ferroptosis-, and inflammation-related pathways. Although docking-derived binding energies and interaction patterns cannot be interpreted as evidence of biological activity or therapeutic efficacy, they offered a structure-based framework for prioritizing candidate compound–target relationships for future experimental investigation.

Several limitations should be considered when interpreting these results. First, the primary transcriptomic discovery analysis was based on a single public microarray cohort and therefore requires further validation in larger independent datasets and experimental models. Moreover, detailed patient-level clinical phenotypes were not available in the public transcriptomic dataset, limiting the ability to relate molecular findings to diabetes subtype, disease duration, glycemic control, treatment status, or clinical outcomes. Because T1D- and T2D-associated cardiomyopathy may involve distinct metabolic, inflammatory, and pathophysiological mechanisms, the present findings should not be interpreted as diabetes subtype-specific observations. The relatively small network size and the presence of disconnected nodes also limit the ability to infer robust interaction architecture. In addition, enrichment analyses are inherently dependent on input gene composition and database annotations and may introduce bias toward well-characterized biological processes. Because the ROC and machine-learning analyses were derived from a single discovery cohort, the resulting performance estimates should be interpreted cautiously. Although exploratory sample-level external evaluation was performed in GSE263297, the limited sample size, cohort differences, and platform differences preclude definitive claims of diagnostic validity or clinical utility. Several inflammation- and immune-associated transcripts, including *CD163*, *S100A8*, *PTX3*, and *SERPINE1*, were present among the differentially expressed genes; however, immune cell deconvolution was not performed in the present study because the analysis was based on bulk myocardial microarray data and a heterogeneous cardiomyopathy cohort. Future studies using single-cell transcriptomics, spatial transcriptomics, or validated immune deconvolution frameworks may help clarify whether macrophage-, monocyte-, neutrophil-, T-cell-, or stromal-related signatures contribute to the observed remodeling profile.

Molecular docking analyses represent structure-based computational predictions and do not account for the full complexity of biological systems, including protein dynamics, conformational flexibility, post-translational modifications, tissue-specific context, or in vivo pharmacokinetics and pharmacodynamics. Furthermore, the ACSL4 structure was derived from an AlphaFold model because an experimentally resolved ligand-bound structure was not available. In addition, *GPX4* contains a catalytically important selenocysteine residue that may not be fully represented by standard AutoDock 4.2.6 atom parameterization. Therefore, *GPX4*-related docking results should be interpreted as approximate estimates of ligand–target compatibility and considered with appropriate methodological caution. *SMAD3* (PDB ID: 1MJS) represents an MH2-domain transcription factor complex for which no well-established small-molecule binding pocket has been experimentally validated. Consequently, SMAD3-related docking results should be interpreted with additional caution and regarded primarily as exploratory structural observations. In addition, the particularly low predicted binding energy obtained for the ruxolitinib–*TGFBR1* interaction should be interpreted cautiously, as ruxolitinib is a known JAK1/JAK2 inhibitor rather than a *TGFBR1*-directed ligand and the observed docking score may reflect structural compatibility within the modeled binding region rather than biologically meaningful target selectivity. Accordingly, predicted binding energies and interaction patterns should be interpreted as indicators of potential structural compatibility rather than evidence of biological activity, target modulation, or therapeutic efficacy.

Finally, the present work was designed as an exploratory and hypothesis-generating in silico study integrating transcriptomic profiling, network analysis, machine learning-based prioritization, and molecular docking. Additional validation using independent patient cohorts, molecular dynamics simulations, target-specific functional assays, and appropriate in vitro and in vivo models will be necessary to further evaluate the biological and translational relevance of the reported findings.

## 5. Conclusions

The present study provides an integrated transcriptome-based exploration of the ferroptosis–fibrosis network in cardiomyopathy and its exploratory potential relevance to DCM. Differential expression, enrichment, and PPI analyses collectively highlighted ECM remodeling, fibrosis-associated pathways, and stress-related biological processes as prominent components of the observed transcriptional landscape. Hub gene analysis identified several ECM–associated candidates, including *LUM*, *ASPN*, *COL1A1*, *COL1A2*, and *COL3A1*, which were further prioritized through ROC and machine learning analyses. Complementary machine learning approaches converged on a core signature consisting of *FCN3*, *HOPX*, *CNN1*, and *GLUL*, with *LUM* additionally supported by two independent algorithms. ROC analyses provided exploratory evidence of sample-level separation for several ECM-related transcripts within the discovery cohort, while additional internal cross-validation and exploratory external sample-level evaluation provided non-definitive observations regarding the machine-learning–derived four-gene signature. In addition, molecular docking analyses provided a structure-based framework for examining selected ligand–target combinations across fibrosis-, ferroptosis-, inflammatory signaling-, and cellular stress–related pathways. Taken together, these findings suggest that ECM remodeling and ferroptosis-associated stress responses may represent interconnected components of the observed cardiomyopathy-associated transcriptional landscape. However, because the study was based on retrospective transcriptomic analyses and exploratory computational approaches, the results should be regarded as hypothesis-generating rather than mechanistic or therapeutic evidence. Further validation using independent cohorts, functional experiments, and disease-relevant in vitro and in vivo models will be required to clarify the biological significance and potential translational relevance of the identified genes, pathways, and ligand–target relationships.

## Figures and Tables

**Table 1:** Top differentially expressed genes in GSE5406. Top-ranked differentially expressed genes (DEGs) between nonfailing and cardiomyopathy samples are shown.

Gene Symbol	Gene Title	log2FC	adj. *p*. Val
*MYOT*	Myotilin	2.26	9.45 × 10^−22^
*ASPN*	Asporin	−2.48	9.76 × 10^−22^
*LUM*	Lumican	−2.09	1.69 × 10^−21^
*TUBA3D/TUBA3C*	Tubulin alpha 3d/3c	1.19	9.24 × 10^−20^
*SERPINA3*	Serpin family A member 3	1.34	9.63 × 10^−20^
*IL1RL1*	Interleukin 1 receptor-like 1	1.72	6.29 × 10^−18^
*FCN3*	Ficolin 3	1.27	1.06 × 10^−16^
*HOPX*	HOP homeobox	1.50	3.73 × 10^−13^
*CNN1*	Calponin 1	1.35	3.87 × 10^−13^
*MXRA5*	Matrix remodeling associated 5	−1.60	2.75 × 10^−12^
*MATN2*	Matrilin 2	−1.16	4.71 × 10^−12^
*FKBP5*	FKBP prolyl isomerase 5	1.55	5.91 × 10^−12^
*LMCD1*	LIM and cysteine-rich domains 1	1.13	1.27 × 10^−10^
*GLUL*	Glutamate-ammonia ligase	1.46	1.59 × 10^−10^
*PTN*	Pleiotrophin	−1.21	2.62 × 10^−10^

Positive logFC values indicate higher expression in nonfailing control samples relative to cardiomyopathy samples, whereas negative logFC values indicate higher expression in cardiomyopathy samples relative to controls.

**Table 2:** Cross-dataset directional comparison of concordant genes between GSE5406 and GSE263297. GSE263297 log2 fold-change values were recalculated from normalized expression data by comparing ICM-DM samples (*n* = 7) with donor controls (*n* = 7). Statistical comparisons were performed using Welch’s *t*-test and Benjamini–Hochberg false discovery rate correction. Concordance indicates matching expression direction between datasets.

Gene Symbol	Gene Title	GSE5406 log2FC	GSE263297 log2FC	adj. *p*	Direction
*MAFF*	MAF bZIP transcription factor F	1.28	1.242	0.989	Concordant
*FHL1*	Four and a half LIM domains 1	1.05	0.787	0.989	Concordant
*PTX3*	Pentraxin 3	1.40	0.670	0.989	Concordant
*SERPINE1*	Serpin family E member 1	1.71	0.666	0.989	Concordant
*HBA2*	Hemoglobin subunit alpha 2	−1.54	−0.473	0.989	Concordant
*SLC19A2*	Solute carrier family 19 member 2	1.10	0.448	0.989	Concordant
*PDK4*	Pyruvate dehydrogenase kinase 4	1.22	0.433	0.989	Concordant
*THBS1*	Thrombospondin 1	1.00	0.390	0.989	Concordant
*HSP90AA1*	Heat shock protein HSP 90-alpha	1.14	0.357	0.989	Concordant
*NRAP*	Nebulin-related anchoring protein	1.43	0.297	0.989	Concordant
*LMCD1*	LIM and cysteine-rich domains 1	1.13	0.252	0.989	Concordant
*PALLD*	Palladin, cytoskeletal associated protein	1.18	0.225	0.989	Concordant
*FLNC*	Filamin C	1.09	0.189	0.989	Concordant
*MT1M*	Metallothionein 1M	1.11	0.144	0.989	Concordant
*FKBP5*	FKBP prolyl isomerase 5	1.55	0.096	0.989	Concordant
*GLUL*	Glutamate-ammonia ligase	1.46	0.072	0.989	Concordant
*SLC38A2*	Solute carrier family 38 member 2	1.05	0.063	0.989	Concordant
*CNN1*	Calponin 1	1.35	0.060	0.989	Concordant
*HBB*	Hemoglobin subunit beta	−1.61	−0.042	0.989	Concordant
*CCT2*	Chaperonin containing TCP1 subunit 2	1.29	0.007	0.989	Concordant

Adjusted *p*-values were uniformly 0.989 after Benjamini–Hochberg correction of the tested concordant genes, reflecting high raw *p*-values and absence of statistically significant differential expression in the independent GSE263297 cohort.

**Table 3:** Representative enriched Gene Ontology (GO) biological processes, molecular functions, cellular components, and KEGG and Reactome pathways identified from STRING functional enrichment analysis of the differentially expressed gene set. Gene count represents the number of genes associated with each term, and statistical significance is reported as false discovery rate (FDR)-adjusted *p* values. Terms were selected to represent the predominant biological themes of the network.

Category	Term/Pathway	Gene Count	FDR
GO Biological Process	Response to stress	25	0.0025
GO Biological Process	Response to inorganic substance	10	0.0071
GO Biological Process	Nitric oxide transport	3	0.0071
GO Biological Process	ECM organization	7	0.0256
GO Biological Process	Collagen fibril organization	4	0.0299
GO Biological Process	Hydrogen peroxide catabolic process	3	0.0468
GO Molecular Function	ECM structural constituent	8	1.20 × 10^−5^
GO Molecular Function	Structural molecule activity	13	0.00017
GO Molecular Function	ECM structural constituent conferring tensile strength	4	0.0022
GO Molecular Function	Platelet-derived growth factor binding	3	0.0057
GO Cellular Component	Collagen-containing ECM	16	1.09 × 10^−11^
GO Cellular Component	ECM	17	3.12 × 10^−11^
GO Cellular Component	Fibrillar collagen trimer	4	2.39 × 10^−5^
KEGG Pathway	Protein digestion and absorption	5	0.0025
KEGG Pathway	AGE–RAGE signaling pathway in diabetic complications	4	0.0216
Reactome Pathway	Binding and uptake of ligands by scavenger receptors	7	1.04 × 10^−7^
Reactome Pathway	ECM proteoglycans	6	7.53 × 10^−5^
Reactome Pathway	ECM organization	8	0.00086
Reactome Pathway	Integrin cell surface interactions	5	0.0016
Reactome Pathway	Collagen chain trimerization	4	0.0027

**Table 4:** Topological properties of hub genes within the MCC-derived subnetwork. Degree, betweenness centrality, and closeness centrality were calculated using the Analyze Network tool in Cytoscape.

Rank	Protein Symbol	Description	Degree	Betweenness Centrality	Closeness Centrality
1	COL1A1	Collagen alpha-1(I) chain	6	0.345238	0.875000
2	COL1A2	Collagen alpha-2(I) chain	5	0.059524	0.777778
3	COL3A1	Collagen alpha-1(III) chain	5	0.059524	0.777778
4	LUM	Lumican	4	0.119048	0.700000
5	ASPN	Asporin	4	0.119048	0.700000
6	COL15A1	Collagen alpha-1(XV) chain	3	0.000000	0.583333
7	OGN	Osteoglycin	2	0.011905	0.500000
8	THBS1	Thrombospondin-1	1	0.000000	0.500000
9	HSP90AA1	Heat shock protein HSP 90-alpha	0	0.000000	0.000000
10	HBB	Hemoglobin subunit beta	0	0.000000	0.000000

**Table 5:** Ranking of hub genes based on MCC scores calculated using the cytoHubba plugin in Cytoscape.

Rank	Gene	MCC Score
1	*COL15A1*	0.46346
2	*ASPN*	0.46346
3	*LUM*	0.46346
4	*COL1A2*	0.45378
5	*COL1A1*	0.45378
6	*COL3A1*	0.45378
7	*HBB*	0.30779
8	*HSP90AA1*	0.00000
9	*OGN*	0.00000
10	*THBS1*	0.00000

**Table 6:** Disease enrichment analysis of differentially expressed genes using the Enrichr platform (DisGeNET and OMIM Disease libraries). Terms are ranked according to adjusted *p*-values.

Category	Disease Term	Adjusted *p*-Value	Overlap	Key Genes
DisGeNET	Congestive heart failure	4.63 × 10^−9^	18/884	*NPPA*, *MYH6*, *COL1A1*, *COL3A1*
DisGeNET	Heart failure	7.56 × 10^−9^	17/815	*NPPA*, *MYH6*, *COL1A1*
DisGeNET	Myocardial infarction	3.82 × 10^−6^	15/966	*NPPA*, *PTX3*, *COL1A1*
DisGeNET	Cardiovascular diseases	1.51 × 10^−5^	13/787	*NPPA*, *FLNC*, *PTX3*
DisGeNET	Hypertrophic cardiomyopathy	1.45 × 10^−4^	8/328	*MYH6*, *FLNC*, *NPPA*
DisGeNET	Diabetes mellitus	6.51 × 10^−5^	16/1507	*PDK4*, *S100A8*, *SERPINE1*
DisGeNET	Atherosclerosis	6.51 × 10^−5^	14/1134	*SERPINE1*, *PTX3*
DisGeNET	Fibrosis	2.60 × 10^−4^	6/169	*COL1A1*, *COL3A1*, *COL1A2*
OMIM	Ehlers–Danlos syndrome	2.29 × 10^−5^	3/11	*COL1A1*, *COL3A1*, *COL1A2*
OMIM	Anemia	7.91 × 10^−5^	4/61	*HBA1*, *HBA2*, *HBB*
OMIM	Osteoporosis	1.04 × 10^−3^	2/11	*COL1A1*, *COL1A2*
OMIM	Myopathy	1.27 × 10^−2^	2/44	*FHL1*, *FLNC*
OMIM	Hypertrophic cardiomyopathy	5.78 × 10^−2^	1/17	*MYH6*

**Table 7:** Pathway enrichment analysis of differentially expressed genes using KEGG 2026 and Reactome 2024 databases. Terms are ranked according to adjusted *p*-values.

Category	Pathway	Adjusted *p*-Value	Overlap	Key Genes
KEGG 2026	Cytoskeleton in muscle cells	1.01 × 10^−5^	8/230	*MYH6*, *FLNC*, *COL1A1*
KEGG 2026	Protein digestion and absorption	1.78 × 10^−4^	5/102	*COL1A1*, *COL3A1*, *COL1A2*
KEGG 2026	AGE–RAGE signaling pathway in diabetic complications	2.21 × 10^−3^	4/100	*COL1A1*, *COL3A1*, *SERPINE1*
KEGG 2026	ECM–receptor interaction	1.99 × 10^−2^	3/88	*COL1A1*, *COL1A2*, *THBS1*
KEGG 2026	DCM	1.99 × 10^−2^	4/203	*COL1A1*, *COL3A1*, *PDK4*
KEGG 2026	PI3K–Akt signaling pathway	7.12 × 10^−2^	4/355	*COL1A1*, *HSP90AA1*
Reactome 2024	ECM proteoglycans	6.97 × 10^−6^	6/76	*COL1A1*, *COL3A1*, *LUM*
Reactome 2024	ECM organization	7.64 × 10^−5^	8/300	*COL1A1*, *COL3A1*, *COL1A2*
Reactome 2024	Integrin cell surface interactions	1.79 × 10^−4^	5/85	*COL1A1*, *COL3A1*, *THBS1*
Reactome 2024	Collagen formation	2.10 × 10^−3^	4/90	*COL1A1*, *COL3A1*, *COL1A2*
Reactome 2024	Collagen degradation	7.61 × 10^−4^	4/64	*COL1A1*, *COL3A1*, *COL1A2*
Reactome 2024	Platelet activation, signaling and aggregation	6.40 × 10^−3^	5/262	*SERPINE1*, *THBS1*

**Table 8:** Diagnostic performance of candidate hub genes in the GSE5406 cohort. Receiver operating characteristic (ROC) analysis was performed using normalized gene expression values to assess the discriminatory ability of candidate hub genes between cardiomyopathy and nonfailing myocardial samples.

Gene	Probe ID	AUC	95% CI	Sensitivity	Specificity
*LUM*	201744_s_at	0.960	0.923–0.997	0.907	0.875
*ASPN*	219087_at	0.952	0.889–1.000	0.985	0.875
*COL3A1*	215076_s_at	0.835	0.715–0.954	0.778	0.875
*COL1A1*	202310_s_at	0.832	0.703–0.960	0.876	0.750
*COL1A2*	202404_s_at	0.827	0.705–0.949	0.856	0.812
*THBS1*	201109_s_at	0.802	0.686–0.919	0.871	0.688
*SERPINE1*	202628_s_at	0.780	0.632–0.929	0.856	0.750
*PTX3*	206157_at	0.748	0.615–0.881	0.902	0.562
*S100A8*	202917_s_at	0.714	0.598–0.830	0.644	0.750
*PDK4*	205960_at	0.697	0.532–0.861	0.861	0.562

**Table 9:** Machine learning–based feature selection results in the GSE5406 dataset. Candidate genes were identified using three independent machine learning approaches, including least absolute shrinkage and selection operator (LASSO) regression, random forest classification, and support vector machine–recursive feature elimination (SVM-RFE).

Method	Number of Selected Genes	Selected Genes
LASSO	9	*FCN3*, *HOPX*, *CNN1*, *GLUL*, *NPPA*, *MT1X*, *SLC19A2*, *PTX3*, *COL3A1*
Random Forest	18	*ASPN*, *MXRA5*, *PALLD*, *LUM*, *TUBA3C/TUBA3D*, *GLUL*, *SERPINA3*, *CNN1*, *MYOT*, *MATN2*, *HSP90AA1*, *IL1RL1*, *FCN3*, *HOPX*, *CCT2*, *COL1A2*, *ANKRD2*, *HBB*
SVM-RFE	34	*LUM*, *ASPN*, *FCN3*, *GLUL*, *SERPINA3*, *MXRA5*, *PTN*, *MATN2*, *HOPX*, *PALLD*, *SLC38A2*, *OGN*, *IL1RL1*, *MYOT*, *CNN1*, *FKBP5*, *CCT2*, *NPPA*, *PLIN2*, *MT1X*, *COL1A2*, *CD163*, *COL15A1*, *LYVE1*, *HSP90AA1*, *PLA2G2A*, *TUBA3C/TUBA3D*, *COL3A1*, *SLC19A2*, *FLNC*, *ANKRD2*, *COL1A1*, *MT1M*, *MYH6*

Note: *FCN3*, *HOPX*, *CNN1*, and *GLUL* were consistently identified by all three machine learning algorithms and were therefore considered the core machine learning signature. Genes identified by at least two algorithms included *NPPA*, *MT1X*, *SLC19A2*, *COL3A1*, *ASPN*, *MXRA5*, *PALLD*, *LUM*, *TUBA3C/TUBA3D*, *SERPINA3*, *MYOT*, *MATN2*, *HSP90AA1*, *IL1RL1*, *CCT2*, *COL1A2*, and *ANKRD2*. Feature-selection results should be interpreted as exploratory and hypothesis-generating because model development and feature selection were performed within the same discovery cohort and in the presence of marked class imbalance.

**Table 10:** Exploratory evaluation analyses of the machine-learning–derived four-gene signature. Validation was performed at three levels, including internal cross-validation within the discovery cohort, cross-cohort comparison of differential-expression patterns, and exploratory sample-level external evaluation in an independent cohort. (**A**) Internal validation using stratified class-weighted five-fold cross-validation. (**B**) Cross-cohort comparison between GSE5406 and GSE263297. (**C**) Exploratory sample-level external evaluation in GSE263297.

(**A**)		
Metric	Value	
Cross-validated AUC	0.934	
95% CI lower bound	0.820	
95% CI upper bound	1.000	
Nonfailing control samples	16	
Cardiomyopathy samples	194	
(**B**)		
Metric	Value	
Discovery genes tested	51	
Genes found in independent cohort	51	
Same-direction genes	20	
Directional concordance (%)	39.2	
Same-direction genes with |logFC| ≥ 1	1	
(**C**)		
Model/Gene	AUC	95% CI
Four-gene signature (*FCN3* + *HOPX* + *CNN1* + *GLUL*)	0.673	0.380–0.967
*FCN3*	0.806	0.534–1.000
*HOPX*	0.622	0.369–0.876
*GLUL*	0.582	0.281–0.882
*CNN1*	0.541	0.219–0.862

## Data Availability

Data Availability Statement: The datasets analyzed in this study are publicly available in the NCBI Gene Expression Omnibus (GEO) repository under accession numbers GSE5406 and GSE263297. All data generated during the study are included in this published article and its Supplementary Materials.

## Supplementary Materials

The following supporting information can be downloaded at: https://www.mdpi.com/article/10.3390/biomedicines14071501/s1, Figure S1: OMIM disease enrichment analysis of differentially expressed genes; Figure S2: KEGG pathway–gene association clustergram; Figure S3: Reactome pathway–gene association clustergram; Figure S4: Chemical structures of the candidate compounds included in the molecular docking analyses; Figure S5: 2D protein–ligand interaction diagrams for TGFBR1-, STAT3-, and GPX4-targeted docking simulations; Figure S6: 2D protein–ligand interaction diagrams for TGFBR1-, STAT3-, and GPX4-targeted docking simulations; Figure S7: 2D protein–ligand interaction diagrams for AKT1-, SMAD3-, and ACSL4-targeted docking simulations; Figure S8: 2D protein–ligand interaction diagrams for AKT1-, SMAD3-, and ACSL4-targeted docking simulations. Table S1: Complete list of differentially expressed probe sets identified in the GSE5406 dataset; Table S2: Mechanistically relevant candidate compounds associated with the ferroptosis–fibrosis axis; Table S3: Target proteins and grid parameters used in molecular docking analyses; Table S4: Detailed molecular docking interaction profiles of candidate compounds with fibrosis-, ferroptosis-, inflammatory signaling-, and cellular stress–related target proteins.

## Abbreviations

The following abbreviations are used in this manuscript:

ACSL4	Acyl-CoA Synthetase Long-Chain Family Member 4
AGE–RAGE	Advanced Glycation End Products–Receptor for Advanced Glycation End Products
AKT1	AKT Serine/Threonine Kinase 1
AUC	Area Under the Curve
DCM	Diabetic Cardiomyopathy
DEG	Differentially Expressed Gene
ECM	Extracellular Matrix
FDR	False Discovery Rate
GEO	Gene Expression Omnibus
GO	Gene Ontology
GPX4	Glutathione Peroxidase 4
ICM-DM	Ischemic Cardiomyopathy with Diabetes Mellitus
JAK	Janus Kinase
KEGG	Kyoto Encyclopedia of Genes and Genomes
LASSO	Least Absolute Shrinkage and Selection Operator
LGA	Lamarckian Genetic Algorithm
logFC	Log Fold Change
MCC	Maximal Clique Centrality
NCBI	National Center for Biotechnology Information
NRF2	Nuclear Factor Erythroid 2–Related Factor 2
OMIM	Online Mendelian Inheritance in Man
PDB	Protein Data Bank
PPI	Protein–Protein Interaction
ROC	Receiver Operating Characteristic
ROS	Reactive Oxygen Species
RMSD	Root Mean Square Deviation
SMAD3	SMAD Family Member 3
STAT3	Signal Transducer and Activator of Transcription 3
STRING	Search Tool for the Retrieval of Interacting Genes/Proteins
SVM-RFE	Support Vector Machine–Recursive Feature Elimination
TGF-β	Transforming Growth Factor Beta
TGFBR1	Transforming Growth Factor Beta Receptor 1
UMAP	Uniform Manifold Approximation and Projection
