# Supplementary material for: An Integrative Transcriptomic, Network Pharmacology, and Molecular Docking Analysis of the Ferroptosis–Fibrosis Axis in Cardiomyopathy with Exploratory Relevance to Diabetic Cardiomyopathy
Source: Biomedicines. 2026 Jul 2;14(7):1501. doi: 10.3390/biomedicines14071501 (PMC13404720; doi:10.3390/biomedicines14071501)
Supplement: Supplementary file 1 [file biomedicines-14-01501-s001.zip › biomedicines-4392934-supplementary.pdf]

Supplementary Materials

An Integrative Transcriptomic, Network Pharmacology, and Molecular Docking Analysis of the Ferroptosis–Fibrosis Axis in Cardiomyopathy with Exploratory Relevance to Diabetic Cardiomyopathy

S1. Overview

The supplementary materials provide additional data related to the transcriptomic, network, enrichment, and molecular docking analyses presented in the main manuscript. These materials include the complete list of differentially expressed genes and detailed visualizations of disease and pathway enrichment analyses. Additional supplementary tables provide mechanistic contextualization of candidate compounds associated with ferroptosis-, fibrosis-, oxidative stress-, and inflammation-related signaling pathways relevant to cardiomyopathy remodeling and with exploratory relevance to diabetic cardiomyopathy. In addition, supplementary molecular docking information is provided in a dedicated section following the enrichment figures, including methodological details, ligand–target interaction tables, and grid parameter settings. The overall structure of the supplementary materials follows the analytical workflow presented in the main manuscript to ensure consistency and clarity.

Supplementary Tables

Table S1. Complete list of differentially expressed probe sets identified in the GSE5406 dataset. Differentially expressed probe sets between nonfailing myocardial samples and cardiomyopathy samples (ischemic and idiopathic combined) were identified using GEO2R analysis based on the limma framework. Probe sets were filtered using a threshold of adjusted p-value < 0.05 and absolute log2 fold-change ≥ 1. The table includes probe identifiers (Probe ID), adjusted p-values (adj.P.Val), raw p-values (P.Value), log2 fold-change (logFC), and corresponding gene symbols and gene titles.

Probe ID	adj.P.Val	P.Value	logFC	Gene Symbol	Gene Title
219728_at	9.45×10^{-22}	1.27×10^{-25}	2.26	*MYOT*	Myotilin
219087_at	9.76×10^{-22}	1.75×10^{-25}	-2.48	*ASPN*	Asporin
201744_s_at	1.69×10^{-21}	3.79×10^{-25}	-2.09	*LUM*	Lumican
216323_x_at	9.24×10^{-20}	2.49×10^{-23}	1.19	*TUBA3D/TUBA3C*	Tubulin alpha 3d/3c
202376_at	9.63×10^{-20}	3.03×10^{-23}	1.34	*SERPINA3*	Serpin family A member 3
207526_s_at	6.29×10^{-18}	2.54×10^{-21}	1.72	*IL1RL1*	Interleukin 1 receptor-like 1
205866_at	1.06×10^{-16}	5.22×10^{-20}	1.27	*FCN3*	Ficolin 3
211597_s_at	3.73×10^{-13}	2.85×10^{-16}	1.50	*HOPX*	HOP homeobox
203951_at	3.87×10^{-13}	3.13×10^{-16}	1.35	*CNN1*	Calponin 1
209596_at	2.75×10^{-12}	2.71×10^{-15}	-1.60	*MXRA5*	Matrix remodeling associated 5
202350_s_at	4.71×10^{-12}	5.50×10^{-15}	-1.16	*LOC100506558/MATN2*	LOC100506558 / Matrilin 2
204560_at	5.91×10^{-12}	7.16×10^{-15}	1.55	*FKBP5*	FKBP prolyl isomerase 5

218574_s_at	1.27×10^{-10}	1.99×10^{-13}	1.13	*LMCD1*	LIM and cysteine-rich domains 1
200648_s_at	1.59×10^{-10}	2.85×10^{-13}	1.46	*GLUL*	Glutamate-ammonia ligase
211737_x_at	2.62×10^{-10}	4.94×10^{-13}	-1.21	*PTN*	Pleiotrophin
217202_s_at	3.68×10^{-10}	8.59×10^{-13}	1.45	*GLUL*	Glutamate-ammonia ligase
221872_at	5.34×10^{-10}	1.34×10^{-12}	1.20	*RARRES1*	Retinoic acid receptor responder 1
207876_s_at	1.52×10^{-9}	4.58×10^{-12}	1.09	*FLNC*	Filamin C
201946_s_at	4.75×10^{-9}	1.64×10^{-11}	1.29	*CCT2*	Chaperonin containing TCP1 subunit 2
220924_s_at	1.29×10^{-8}	5.28×10^{-11}	1.05	*SLC38A2*	Solute carrier family 38 member 2
210096_at	1.62×10^{-8}	7.25×10^{-11}	1.35	*CYP4B1*	Cytochrome P450 family 4 subfamily B member 1
209957_s_at	4.62×10^{-8}	2.37×10^{-10}	-1.86	*NPPA*	Natriuretic peptide A
218041_x_at	7.29×10^{-8}	3.96×10^{-10}	1.04	*SLC38A2*	Solute carrier family 38 member 2
202627_s_at	7.45×10^{-8}	4.11×10^{-10}	1.28	*SERPINE1*	Serpin family E member 1
209122_at	1.68×10^{-7}	1.05×10^{-9}	1.10	*PLIN2*	Perilipin 2
203477_at	1.83×10^{-7}	1.18×10^{-9}	-1.06	*COL15A1*	Collagen type XV alpha 1 chain
221232_s_at	2.49×10^{-7}	1.71×10^{-9}	1.45	*ANKRD2*	Ankyrin repeat domain 2
204326_x_at	4.69×10^{-7}	3.62×10^{-9}	1.14	*MT1X*	Metallothionein 1X
201109_s_at	6.12×10^{-7}	5.00×10^{-9}	1.00	*THBS1*	Thrombospondin 1
202628_s_at	6.12×10^{-7}	5.00×10^{-9}	1.71	*SERPINE1*	Serpin family E member 1
200906_s_at	9.12×10^{-7}	7.90×10^{-9}	1.18	*PALLD*	Palladin, cytoskeletal associated protein
211696_x_at	1.64×10^{-6}	1.65×10^{-8}	-1.47	*HBB*	Hemoglobin subunit beta
209116_x_at	1.64×10^{-6}	1.65×10^{-8}	-1.61	*HBB*	Hemoglobin subunit beta
209681_at	1.73×10^{-6}	1.79×10^{-8}	1.10	*SLC19A2*	Solute carrier family 19-member 2
202403_s_at	1.73×10^{-6}	1.79×10^{-8}	-1.12	*COL1A2*	Collagen type I alpha 2 chain
214414_x_at	2.12×10^{-6}	2.32×10^{-8}	-1.54	*HBA2/HBA1*	Hemoglobin subunit alpha 2/1
217414_x_at	2.55×10^{-6}	2.93×10^{-8}	-1.37	*HBA2/HBA1*	Hemoglobin subunit alpha 2/1
218730_s_at	3.00×10^{-6}	3.63×10^{-8}	-1.01	*OGN*	Osteoglycin
220037_s_at	3.21×10^{-6}	3.91×10^{-8}	1.14	*LYVE1*	Lymphatic vessel endothelial hyaluronan receptor 1
203645_s_at	4.43×10^{-6}	5.74×10^{-8}	1.46	*CD163*	CD163 molecule
215049_x_at	4.85×10^{-6}	6.47×10^{-8}	1.45	*CD163*	CD163 molecule
203649_s_at	7.41×10^{-6}	1.06×10^{-7}	1.32	*PLA2G2A*	Phospholipase A2 group IIA
217546_at	8.50×10^{-6}	1.26×10^{-7}	1.11	*MT1M*	Metallothionein 1M
211968_s_at	9.27×10^{-6}	1.39×10^{-7}	1.14	*HSP90AA1*	Heat shock protein 90 alpha family class A member 1
211745_x_at	1.33×10^{-5}	2.18×10^{-7}	-1.35	*HBA2/HBA1*	Hemoglobin subunit alpha 2/1
211969_at	1.86×10^{-5}	3.35×10^{-7}	1.09	*HSP90AA1*	Heat shock protein 90 alpha family class A member 1
204018_x_at	2.02×10^{-5}	3.71×10^{-7}	-1.26	*HBA2/HBA1*	Hemoglobin subunit alpha 2/1
206157_at	2.94×10^{-5}	5.80×10^{-7}	1.40	*PTX3*	Pentraxin 3
211699_x_at	4.04×10^{-5}	8.44×10^{-7}	-1.23	*HBA2/HBA1*	Hemoglobin subunit alpha 2/1
214468_at	4.71×10^{-5}	1.00×10^{-6}	1.40	*MYH6*	Myosin heavy chain 6
36711_at	5.40×10^{-5}	1.19×10^{-6}	1.28	*MAFF*	MAF bZIP transcription factor F
217232_x_at	1.16×10^{-4}	2.97×10^{-6}	-1.16	*HBB*	Hemoglobin subunit beta
209458_x_at	1.21×10^{-4}	3.13×10^{-6}	-1.20	*HBA2/HBA1*	Hemoglobin subunit alpha 2/1
202310_s_at	1.77×10^{-4}	4.95×10^{-6}	-1.41	*COL1A1*	Collagen type I alpha 1 chain
215076_s_at	1.78×10^{-4}	4.99×10^{-6}	-1.15	*COL3A1*	Collagen type III alpha 1 chain
211161_s_at	1.88×10^{-4}	5.38×10^{-6}	-1.32	*COL3A1*	Collagen type III alpha 1 chain
202404_s_at	1.06×10^{-3}	4.40×10^{-5}	-1.20	*COL1A2*	Collagen type I alpha 2 chain
207089_at	2.05×10^{-3}	9.97×10^{-5}	1.43	*NRAP*	Nebulin-related anchoring protein

201539_s_at	3.15×10^{-3}	1.69×10^{-4}	1.05	*FHL1*	Four and a half LIM domains 1
204409_s_at	1.06×10^{-2}	8.20×10^{-4}	-1.51	*EIF1AY*	Eukaryotic translation initiation factor 1A, Y-linked
205960_at	1.75×10^{-2}	1.62×10^{-3}	1.22	*PDK4*	Pyruvate dehydrogenase kinase 4
201909_at	2.07×10^{-2}	2.02×10^{-3}	-1.16	*RPS4Y1*	Ribosomal protein S4, Y-linked 1
202917_s_at	4.52×10^{-2}	5.73×10^{-3}	1.03	*S100A8*	S100 calcium binding protein A8

For visualization purposes, logFC values are color-coded, with positive values shown in red and negative values shown in blue.

Table S2. Mechanistically relevant candidate compounds associated with the ferroptosis–fibrosis axis.

Compound	Primary target	Mechanistic relevance
Finerenone	Mineralocorticoid receptor	Anti-fibrotic and anti-inflammatory signaling modulation associated with cardiac remodeling
Bardoxolone methyl	NRF2 / oxidative stress signaling	Modulation of oxidative stress and inflammatory signaling pathways associated with fibrosis and ferroptosis-related injury
Ruxolitinib	JAK1/JAK2 signaling	Modulation of inflammatory and cytokine-associated signaling pathways potentially linked to fibrosis progression and ferroptosis-associated stress responses
Pirfenidone	TGF-β / fibrotic signaling	Antifibrotic modulation relevant to extracellular matrix remodeling and myocardial fibrosis
Deferiprone	Iron chelation / iron-handling axis	Iron chelation potentially relevant to iron-dependent oxidative injury and ferroptosis-related stress
Liproxstatin-1	Ferroptosis / lipid peroxidation inhibition	Experimental ferroptosis inhibitor used as a representative ferroptosis-modulating compound relevant to lipid peroxidation–associated oxidative injury.

Supplementary Figures

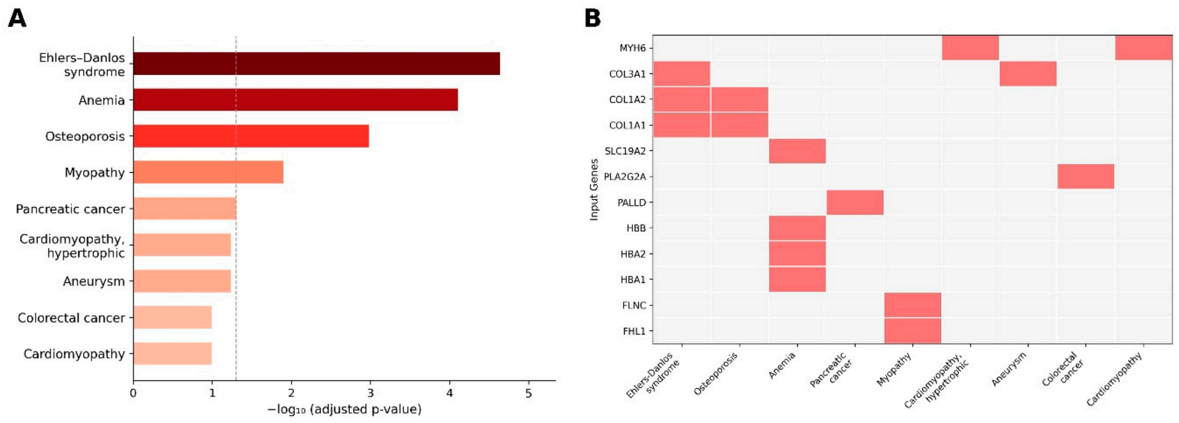

Figure S1. OMIM disease enrichment analysis of differentially expressed genes. (A) Bar plot showing the top enriched disease terms identified from the OMIM database, ranked according to adjusted p-values. Enriched terms include connective tissue disorders, anemia-related conditions, and selected cardiomyopathy-related phenotypes. (B) Clustergram illustrating gene–disease associations within the OMIM dataset. Collagen-related genes (*COL1A1, COL1A2, COL3A1*) are primarily associated with connective tissue disorders, while hemoglobin-related genes (*HBA1, HBA2, HBB*) are linked to anemia-related conditions. These results provide complementary support to the DisGeNET-based findings and are interpreted as exploratory.

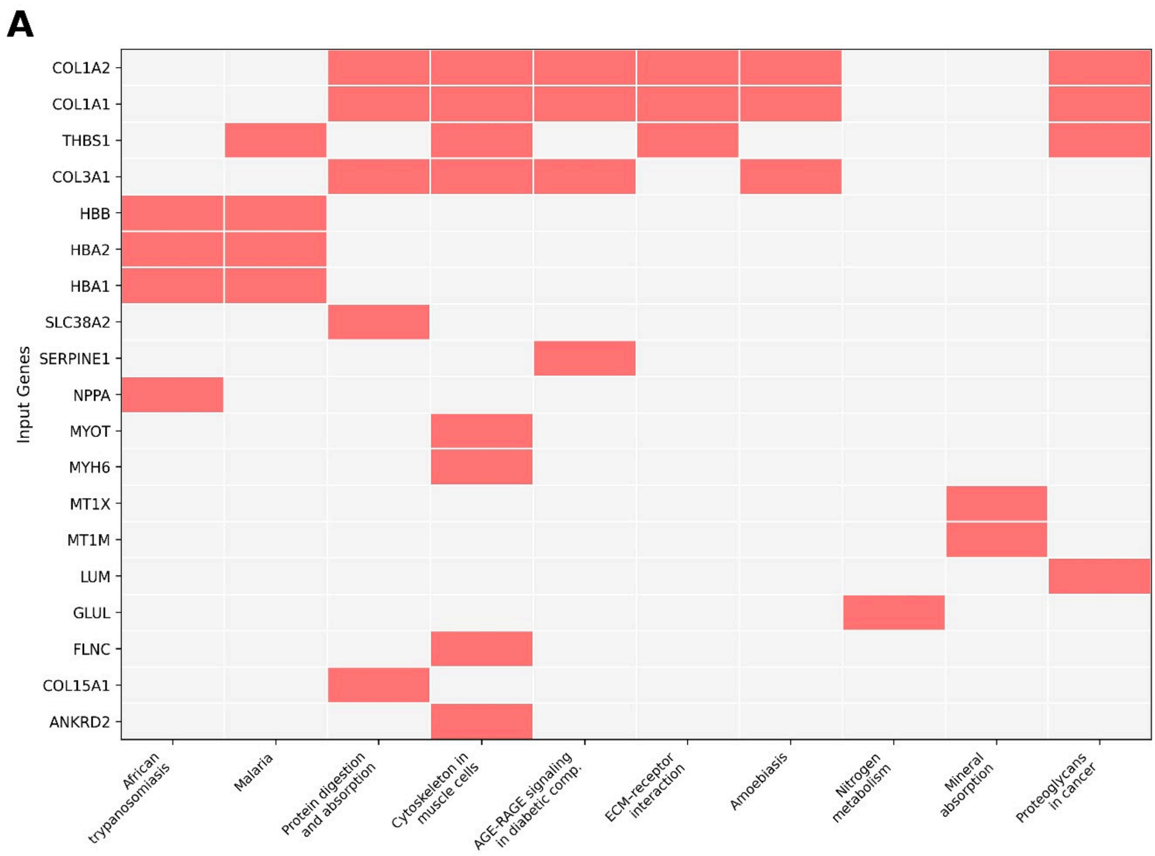

Figure S2. KEGG pathway–gene association clustergram. Clustergram showing the relationships between enriched KEGG pathways and input genes. Pathways include cytoskeleton organization, extracellular matrix (ECM)–receptor interaction, AGE–RAGE signaling in diabetic complications, and protein digestion and absorption. The visualization highlights the contribution of collagen-related and structural genes to multiple enriched pathways. These patterns are descriptive and support the pathway enrichment results presented in the main text.

A

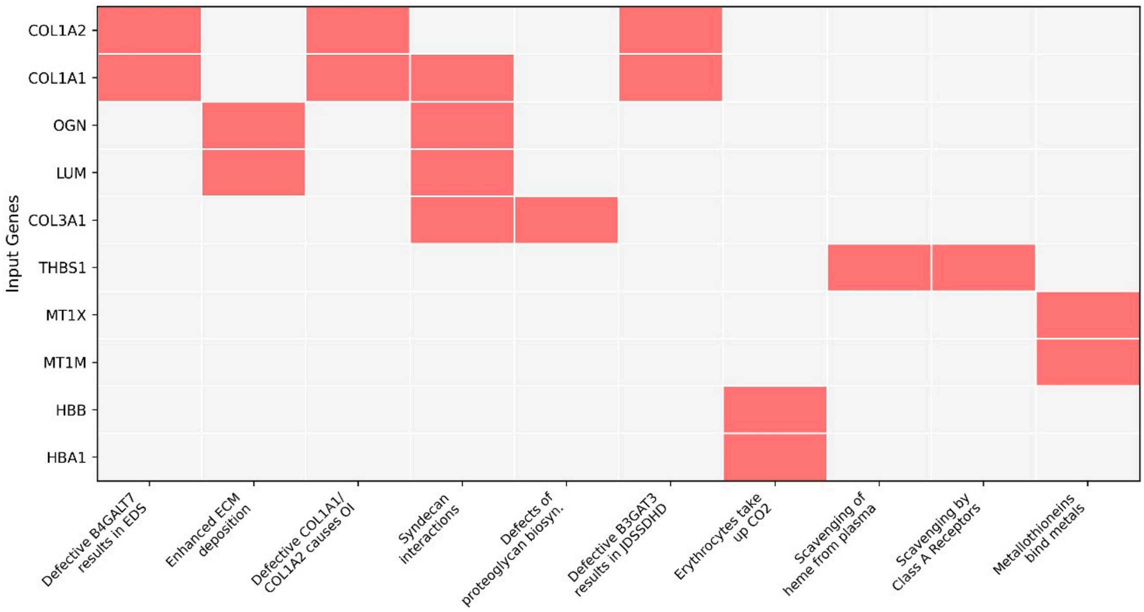

Figure S3. Reactome pathway–gene association clustergram. Clustergram depicting the association between enriched Reactome pathways and input genes. Prominent pathways include extracellular matrix organization, ECM proteoglycans, collagen formation and degradation, and integrin-mediated interactions. The clustering pattern underscores the involvement of extracellular matrix–related processes and main pathway enrichment analysis from the main pathway enrichment analysis. These observations are presented as hypothesis-generating.

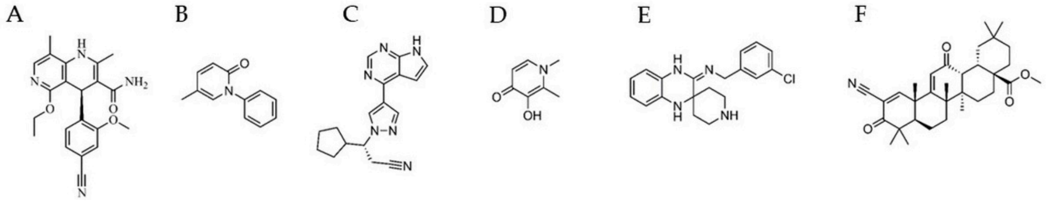

Figure S4. Chemical structures of the candidate compounds included in the molecular docking analyses. (A) Finerenone, (B) Pirfenidone, (C) Ruxolitinib, (D) Deferiprone, (E) Liproxstatin-1, and (F) Bardoxolone methyl. These compounds were selected to represent complementary biological processes associated with fibrosis, ferroptosis, oxidative stress, inflammatory signaling, and cellular stress adaptation. Structures are shown for reference and compound identification purposes.

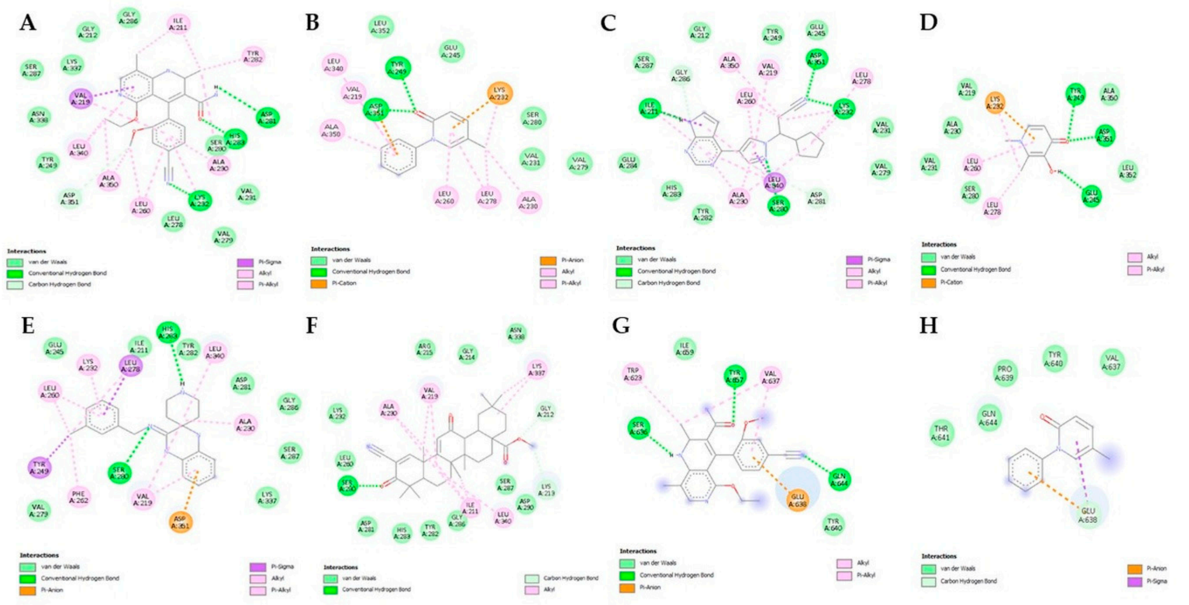

Figure S5. 2D protein–ligand interaction diagrams for TGFBR1-, STAT3-, and GPX4-targeted docking simulations. (A) Finerenone–TGFBR1, (B) Pirfenidone–TGFBR1, (C) Ruxolitinib–TGFBR1, (D) Deferiprone–TGFBR1, (E) Liproxstatin-1–TGFBR1, (F) Bardoxolone methyl–TGFBR1, (G) Finerenone–STAT3, (H) Pirfenidone–STAT3. Interaction diagrams were generated using BIOVIA Discovery Studio Visualizer and are presented to illustrate residue-level interaction patterns within the predicted binding regions. These visualizations are intended for exploratory structural interpretation and should not be considered evidence of biological activity or target inhibition.

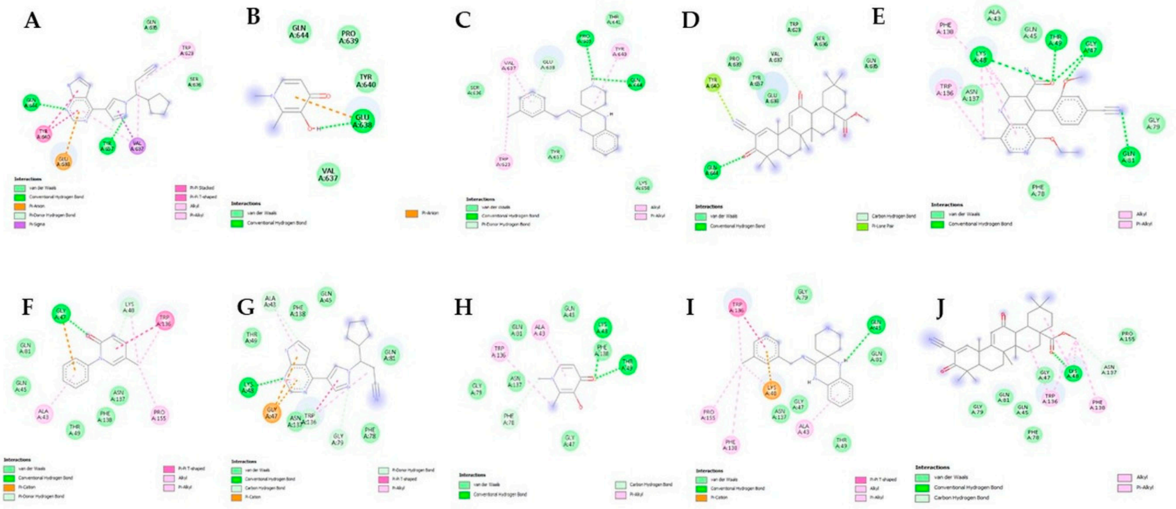

Figure S6. 2D protein–ligand interaction diagrams for TGFBR1-, STAT3-, and GPX4-targeted docking simulations. (A) Ruxolitinib–STAT3, (B) Deferiprone–STAT3, (C) Liproxstatin-1–STAT3, (D) Bardoxolone methyl–STAT3, (E) Finerenone–GPX4, (F) Pirfenidone–GPX4, (G) Ruxolitinib–GPX4, (H) Deferiprone–GPX4, (I) Liproxstatin-1–GPX4, and (J) Bardoxolone methyl–GPX4. Interaction diagrams were generated using BIOVIA Discovery Studio Visualizer and are presented to illustrate residue-level interaction patterns within the predicted binding regions. These visualizations are intended for exploratory structural interpretation and should not be considered evidence of biological activity or target inhibition.

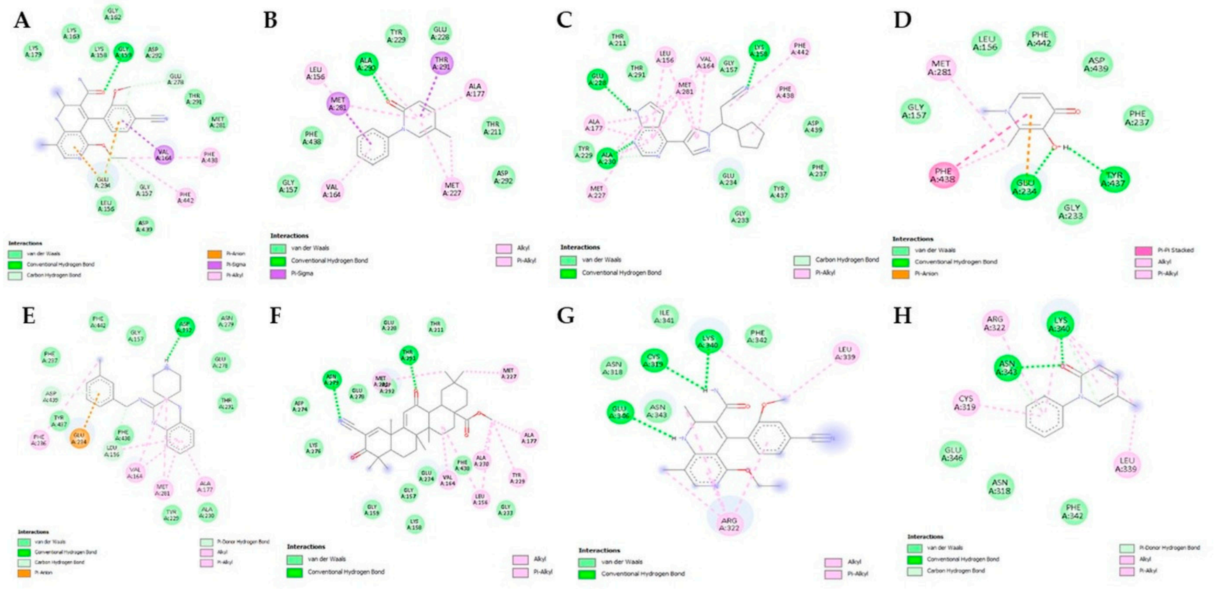

Figure S7. 2D protein–ligand interaction diagrams for AKT1-, SMAD3-, and ACSL4-targeted docking simulations. (A) Finerenone–AKT1, (B) Pirfenidone–AKT1, (C) Ruxolitinib–AKT1, (D) Deferiprone–AKT1, (E) Liproxstatin-1–AKT1, (F) Bardoxolone methyl–AKT1, (G) Finerenone–SMAD3, (H) Pirfenidone–SMAD3. Interaction diagrams were generated using BIOVIA Discovery Studio Visualizer and are presented to facilitate visualization of residue-level interactions and ligand orientation within the predicted binding pockets. Findings should be interpreted as exploratory computational observations within the overall docking framework.

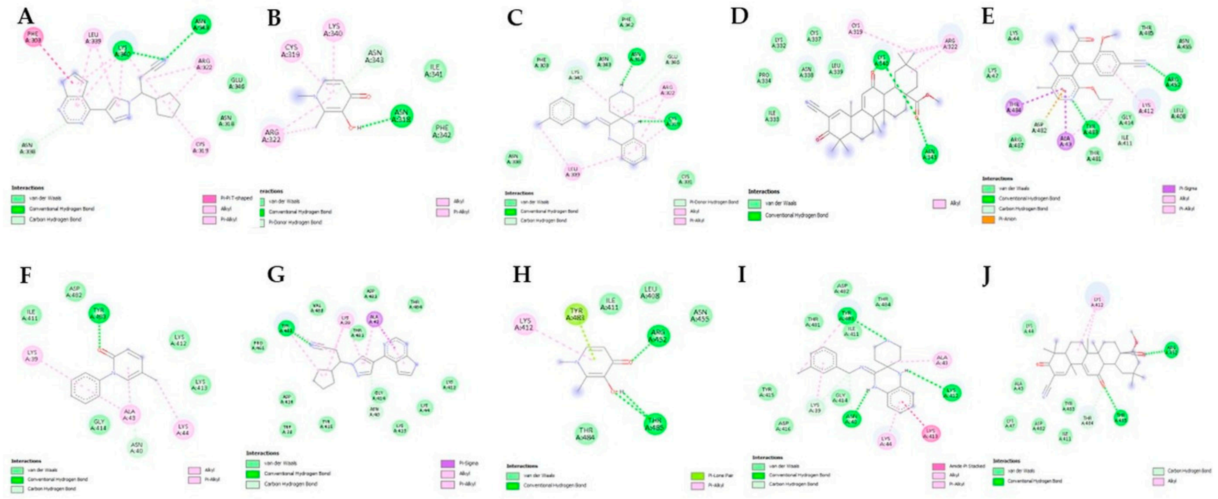

Figure S8. 2D protein–ligand interaction diagrams for AKT1-, SMAD3-, and ACSL4-targeted docking simulations. (A) Ruxolitinib–SMAD3, (B) Deferiprone–SMAD3, (C) Liproxstatin-1–SMAD3, (D) Bardoxolone methyl–SMAD3, (E) Finerenone–ACSL4, (F) Pirfenidone–ACSL4, (G) Ruxolitinib–ACSL4, (H) Deferiprone–ACSL4, (I) Liproxstatin-1–ACSL4, and (J) Bardoxolone methyl–ACSL4. Interaction diagrams were generated using BIOVIA Discovery Studio Visualizer and are presented to facilitate visualization of residue-level interactions and ligand orientation within the predicted binding pockets. Findings should be interpreted as exploratory computational observations within the overall docking framework.

S2. Supplementary Molecular Docking Methods

S2.1. Software and Computational Environment

Molecular docking simulations were performed using AutoDock 4.2.6 with the Lamarckian Genetic Algorithm. Ligand and receptor preparation, grid box definition, and docking file generation were conducted using AutoDockTools (MGLTools v1.5.6). Ligand structure generation and energy minimization were performed using ChemDraw Ultra 12.0 and ChemBio3D Ultra 13.0 with the MMFF94 force field. File format conversions were carried out using Open Babel v3.1.1. Post-docking visualization and residue-level interaction analyses were performed using BIOVIA Discovery Studio Visualizer 2025 Client and PyMOL v2.5.

S2.2. Ligand Preparation

The chemical structures of the selected ligands were initially generated using ChemDraw Ultra 12.0. Three-dimensional conformations were subsequently produced in ChemBio3D Ultra 13.0 and subjected to energy minimization using the Merck Molecular Force Field 94 (MMFF94). Energy-minimized conformers were saved in PDB format prior to docking preparation.

Ligand structures were converted into PDBQT format using Open Babel v3.1.1. During ligand preparation, Gasteiger charges were assigned, polar hydrogen atoms were added, and rotatable bonds were defined according to AutoDock requirements. Ligand preparation procedures were applied consistently across all compounds to preserve methodological comparability among docking simulations.

S2.3. Receptor Preparation

Experimentally resolved protein structures were retrieved from the Protein Data Bank (PDB), whereas ACSL4 was evaluated using the AlphaFold structural model AF-O60488 because experimentally resolved ligand-bound structures for this target remain limited. Receptor preparation included removal of crystallographic water molecules and non-essential heteroatoms, removal of co-crystallized ligands when applicable, addition of polar hydrogen atoms, and assignment of Kollman charges using AutoDockTools. All receptor structures were converted into PDBQT format prior to docking simulations. For experimentally resolved protein structures containing co-crystallized ligands, grid boxes were preferentially centered on ligand-associated binding regions to preserve structural relevance and improve active-site localization. For ACSL4, predicted binding regions were used for exploratory pocket assessment. Accordingly, docking findings associated with ACSL4 were interpreted cautiously as exploratory and hypothesis-generating structural observations rather than definitive evidence of ligand binding.

S2.4. Docking Protocol

Docking simulations were performed using the Lamarckian Genetic Algorithm implemented in AutoDock 4.2.6. For each ligand–protein pair, 40 independent docking runs were conducted to sample multiple potential binding conformations. Grid maps were generated using AutoDockTools with a grid spacing of 0.375 Å. Docked conformations were evaluated according to predicted binding energy values (ΔG), root mean square deviation (RMSD) values, clustering behavior, and structural compatibility within the predicted binding regions. Binding poses with RMSD values ≤ 2.0 Å were considered structurally consistent and reproducible docking conformations. Representative poses were selected based on low binding energy, cluster consistency, and biologically plausible ligand orientation within the binding pocket.

S2.5. Docking Targets and Grid Parameters

Docking analyses were performed for TGFBR1, SMAD3, STAT3, AKT1, GPX4, and ACSL4, representing profibrotic signaling, cellular stress adaptation, inflammatory signaling, ferroptosis, and redox homeostasis pathways relevant to cardiomyopathy remodeling and with exploratory relevance to diabetic cardiomyopathy. Grid dimensions were standardized as 40 × 40 × 40 Å to preserve methodological comparability across targets, whereas grid center coordinates were optimized individually according to the relevant binding region of each target protein.

Table S3. Target proteins and grid parameters used in molecular docking analyses.

Target protein	Structure source	Grid center x	Grid center y	Grid center z	Grid size
TGFBR1	PDB ID: 1PY5	3.222	9.194	4.417	40 × 40 × 40 Å
SMAD3	PDB ID: 1MJS	25.611	0.833	-9.583	40 × 40 × 40 Å
STAT3	PDB ID: 6NJS	13.000	54.500	0.220	40 × 40 × 40 Å
AKT1	PDB ID: 3MV5	5.083	3.917	16.639	40 × 40 × 40 Å
GPX4	PDB ID: 6HKQ	-24.194	9.944	2.333	40 × 40 × 40 Å
ACSL4	AlphaFold: AF-O60488	13.056	18.444	-6.806	40 × 40 × 40 Å

Because experimentally resolved ligand-bound crystal structures for ACSL4 remain limited, an AlphaFold-predicted structural model was used for exploratory docking analyses. Potential ligand-binding pockets were identified using the CB-Dock2 cavity-detection algorithm applied to the AlphaFold-predicted ACSL4 structure (AF-O60488). The highest-ranked cavity proposed by CB-Dock2 was selected as the docking region, and grid box coordinates were defined based on the geometric center of the predicted pocket. Therefore, ACSL4-related docking findings were interpreted cautiously as exploratory and hypothesis-generating structural observations.

S2.6. Interaction Analysis

Docked protein–ligand complexes were analyzed using BIOVIA Discovery Studio Visualizer and PyMOL. Residue-level interaction analyses included conventional hydrogen bonds, π-cation/π-anion interactions, alkyl/π-alkyl contacts, and π-sigma interactions. Binding poses were evaluated according to structural compatibility within the predicted binding regions together with the overall non-covalent interaction profile. Two-dimensional (2D) interaction diagrams and three-dimensional (3D) binding conformations were generated for structural interpretation of docking poses within an exploratory computational framework.

Table S4. Detailed molecular docking interaction profiles of candidate compounds with fibrosis-, ferroptosis-, inflammatory signaling-, and cellular stress–related target proteins. Predicted binding energies (ΔG, kcal/mol), RMSD values, and residue-level interaction profiles obtained from AutoDock 4.2.6 docking simulations are shown for all analyzed ligand–target pairs. Interaction categories include conventional hydrogen bonds, π-cation/π-anion interactions, alkyl/π-alkyl contacts, and π-sigma interactions. Docking results were interpreted as exploratory computational indicators of structural compatibility and potential ligand–target binding tendencies rather than direct evidence of biological activity, target inhibition, or therapeutic efficacy.

Compound	Binding energy (kcal/mol)	RMSD	Conventional Hydrogen Bond	Pi-cation/ Pi-anion	Alkyl/ pi-Alkyl	Pi-sigma	Target protein	PDB ID
Finerenone	-10.40	0.87	His283<br>Asp281	-	Ile211<br>Val219<br>Ala230<br>Leu260<br>Tyr282<br>Leu340<br>Ala350	Val219	TGFBR1	1PY5
Pirfenidone	-7.23	0.04	Tyr249<br>Asp351	Lys232	Val219<br>Leu260<br>Leu278<br>Ala230<br>Leu340<br>Ala350	-	TGFBR1	1PY5
Ruxolitinib	-12.08	0.09	Ile211<br>Lys232<br>Asp351	-	Val219<br>Ala230<br>Leu260<br>Leu278<br>Ala350	-	TGFBR1	1PY5

Deferiprone	-6.86	0.10	Glu245 Tyr249 Asp351	Lys232	Leu260 Leu248	-	TGFBR1	1PY5
Liproxstatin-1	-11.39	0.03	His283 Ser280	Asp351	Val219 Ala230 Lys232 Leu340 Leu260 Phe262	Tyr249 Leu278	TGFBR1	1PY5
Bardoxolone methyl	-5.25	0.02	Ser280	-	Ile211 Val219 Ala230 Lys337 Leu340	-	TGFBR1	1PY5
Finerenone	-8.49	0.12	Ser636 Tyr657	Glu638	Trp623 Val637	-	STAT3	6NJS
Pirfenidone	-5.74	0.08	-	Glu638	-	Glu638	STAT3	6NJS
Ruxolitinib	-8.59	0.11	Gln644 Tyr657	Glu638	Trp623 Val627	Val637	STAT3	6NJS
Deferiprone	-5.40	0.04	Glu638	Glu638	-	-	STAT3	6NJS
Liproxstatin-1	-9.03	0.21	Pro639 Gln644	-	Trp623 Val637 Tyr640	-	STAT3	6NJS
Bardoxolone methyl	-8.69	0.26	Gln644	-	-	-	STAT3	6NJS
Finerenone	-7.19	0.14	Gly47 Lys48 Thr49 Gln81	-	Trp136 Phe138	-	GPX4	6HKQ

Pirfenidone	-6.63	0.72	Gly47	Gly47	Ala43 Pro155	-	GPX4	6HKQ
Ruxolitinib	-8.06	0.34	Lys48	Gly47	Trp136	-	GPX4	6HKQ
Deferiprone	-5.68	0.06	Lys48 Thr49	-	Ala43 Trp136	-	GPX4	6HKQ
Liproxstatin-1	-7.65	0.29	Gln45	Lys48	Ala43 Phe138 Pro155	-	GPX4	6HKQ
Bardoxolone methyl	-6.58	0.04	Lys48	-	Trp136 Phe138	-	GPX4	6HKQ
Finerenone	-8.60	0.22	Gly159	Glu234	Phe438 Phe442	Val164	AKT1	3MV5
Pirfenidone	-6.71	0.02	Ala230	Met281 Thr291	Leu156 Val164 Ala177 Met227 Ala230	-	AKT1	3MV5
Ruxolitinib	-11.72	0.15	Lys158 Glu228	-	Leu156 Val164 Ala177 Met227 Met281 Phe438 Phe442	-	AKT1	3MV5
Deferiprone	-6.68	0.04	Glu234 Tyr437	-	Met281 Phe438	-	AKT1	3MV5
Liproxstatin-1	-10.10	0.04	Asp292	Glu234	Val164 Ala177 Phe236 Met281	-	AKT1	3MV5

Bardoxolone methyl	-8.75	0.08	Asn279<br>Thr291	-	Leu156<br>Val164<br>Ala177<br>Met227<br>Tyr229<br>Ala230	-	AKT1	3MV5
Finerenone	-7.18	0.27	Cys319<br>Lys340<br>Glu346	-	Arg322<br>Leu339	-	SMAD3	1MJS
Pirfenidone	-5.47	0.05	Lys340<br>Asn343	-	Cys319<br>Arg322<br>Leu339	-	SMAD3	1MJS
Ruxolitinib	-7.84	1.36	Lys340<br>Asn343	-	Cys319<br>Arg322<br>Leu339	-	SMAD3	1MJS
Deferiprone	-5.17	0.03	Asn318	-	Cys319<br>Arg322<br>Lys340	-	SMAD3	1MJS
Liproxstatin-1	-8.55	0.18	Asn318<br>Cys319	-	Arg322<br>Leu339	-	SMAD3	1MJS
Bardoxolone methyl	-6.11	0.21	Lys340<br>Asn343	-	Cys319<br>Arg322	-	SMAD3	1MJS
Finerenone	-10.20	0.42	Arg452<br>Tyr483	Asp482	Ala43<br>Lys412<br>Tyr483	Ala43<br>Thr484	ACSL4	AF-O60488
Pirfenidone	-6.69	0.04	Tyr483	-	Lys39<br>Ala43<br>Lys44	-	ACSL4	AF-O60488
Ruxolitinib	-10.75	0.75	Tyr483	-	Lys39<br>Ala43	Ala43	ACSL4	AF-O60488

Deferiprone	-6.09	0.09	Arg452<br>Thr485	-	Lys412	-	ACSL4	AF-O60488
Liproxstatin-1	-10.86	0.17	Asn40<br>Tyr483<br>Lys412	-	Lys39<br>Ala43<br>Lys44<br>Tyr483	-	ACSL4	AF-O60488
Bardoxolone methyl	-7.72	0.15	Arg452<br>Thr485	-	Lys412	-	ACSL4	AF-O60488

S2.7. Interpretation Framework and Methodological Considerations

Molecular docking findings were interpreted within a conservative and exploratory computational framework. Predicted binding energies, RMSD values, and residue-level interaction patterns were not considered direct evidence of biological activity, functional inhibition, or clinical efficacy. Instead, these parameters were used as computational indicators of structural compatibility and potential ligand–target binding tendencies. Because docking analyses are based on static protein structures or predicted structural models, they do not fully account for protein flexibility, dynamic conformational changes, intracellular biological complexity, or in vivo pharmacokinetic and pharmacodynamic conditions. This limitation was considered particularly relevant for targets with potential conformational flexibility and for ACSL4, which was evaluated using an AlphaFold-predicted structural model due to limited experimentally resolved ligand-bound structural data. Accordingly, docking results were considered exploratory, and hypothesis-generating structural observations. The findings were intended to complement the transcriptomic, enrichment, and network-based analyses rather than to establish mechanistic causality or therapeutic efficacy.

S2.8. Reproducibility Statement

All ligand and receptor preparation steps, grid definitions, docking parameters, and pose selection criteria were applied consistently across the analyzed ligand–target pairs. Docking simulations were performed using the specified software versions and publicly available structural data sources described above. Grid parameters for each target protein are provided in Table S3 to support reproducibility. No post hoc optimization was performed to selectively improve individual ligand–target docking outcomes. The workflow was designed to provide a standardized and reproducible

exploratory docking framework for comparing potential structural compatibility across the selected ferroptosis-, fibrosis-, inflammatory signaling-, oxidative stress-, and cellular survival-related targets.
